# Prediction of Probable Major Depressive Disorder in the Taiwan Biobank: An Integrated Machine Learning and Genome-Wide Analysis Approach

**DOI:** 10.3390/jpm11070597

**Published:** 2021-06-24

**Authors:** Eugene Lin, Po-Hsiu Kuo, Wan-Yu Lin, Yu-Li Liu, Albert C. Yang, Shih-Jen Tsai

**Affiliations:** 1 Department of Biostatistics, University of Washington, Seattle, WA 98195, USA; 2 Department of Electrical & Computer Engineering, University of Washington, Seattle, WA 98195, USA; 3 Graduate Institute of Biomedical Sciences, China Medical University, Taichung 40402, Taiwan; 4 Department of Public Health, Institute of Epidemiology and Preventive Medicine, National Taiwan University, Taipei 10617, Taiwan; phkuo@ntu.edu.tw (P.-H.K.); linwy@ntu.edu.tw (W.-Y.L.); 5 Center for Neuropsychiatric Research, National Health Research Institutes, Miaoli County 35053, Taiwan; ylliou@nhri.org.tw; 6 Division of Interdisciplinary Medicine and Biotechnology, Beth Israel Deaconess Medical Center/Harvard Medical School, Boston, MA 02215, USA; accyang@gmail.com; 7 Institute of Brain Science, National Yang Ming Chiao Tung University, Taipei 112304, Taiwan; 8 Department of Psychiatry, Taipei Veterans General Hospital, Taipei 11217, Taiwan; 9 Division of Psychiatry, National Yang Ming Chiao Tung University, Taipei 112304, Taiwan

**Keywords:** genome-wide association study, machine learning, major depressive disorder, personalized medicine, single nucleotide polymorphisms

## Abstract

In light of recent advancements in machine learning, personalized medicine using predictive algorithms serves as an essential paradigmatic methodology. Our goal was to explore an integrated machine learning and genome-wide analysis approach which targets the prediction of probable major depressive disorder (MDD) using 9828 individuals in the Taiwan Biobank. In our analysis, we reported a genome-wide significant association with probable MDD that has not been previously identified: *FBN1* on chromosome 15. Furthermore, we pinpointed 17 single nucleotide polymorphisms (SNPs) which show evidence of both associations with probable MDD and potential roles as expression quantitative trait loci (eQTLs). To predict the status of probable MDD, we established prediction models with random undersampling and synthetic minority oversampling using 17 eQTL SNPs and eight clinical variables. We utilized five state-of-the-art models: logistic ridge regression, support vector machine, C4.5 decision tree, LogitBoost, and random forests. Our data revealed that random forests had the highest performance (area under curve = 0.8905 ± 0.0088; repeated 10-fold cross-validation) among the predictive algorithms to infer complex correlations between biomarkers and probable MDD. Our study suggests that an integrated machine learning and genome-wide analysis approach may offer an advantageous method to establish bioinformatics tools for discriminating MDD patients from healthy controls.

## 1. Introduction

Significant progress has been made in the interdisciplinary fields of personalized medicine, machine learning, and psychiatry in recent years [1,2,3]. In personalized medicine research, machine learning models have been investigated to develop predictive algorithms that can help facilitate studies of how genetic variants and clinical variables can impact disease status and treatment outcomes in patients [1,2,3]. Advancements in machine learning models have shown promising potential in terms of personalized medicine for patients with psychiatric disorders [1,2,3]. For instance, machine learning models have been employed to derive clinical treatment outcomes in patients with major depressive disorder (MDD) [4,5] as well as disease status in patients with schizophrenia [6,7] using clinical characteristics and genetic variants such as single nucleotide polymorphisms (SNPs). Due to their wide range of potential applications, it has been suggested that machine learning models can play a pivotal role in the future of personalized medicine [8,9,10].

In the arena of personalized medicine, the use of machine learning models to predict disease status in patients with MDD has been a focus of attention. To list several examples, Kessler et al. [11] employed an ensemble regression tree machine learning approach to forecast the persistence and severity of MDD using self-reported survey data (area under the receiver operating characteristic curve (AUC) = 0.71 for forecasting high persistence). Nemesure et al. [12] used an extreme gradient boosting machine learning method to detect MDD using electronic health records (AUC = 0.67). Qi et al. [13] demonstrated an extreme gradient boosting machine learning method to predict the severity of MDD using microRNA expression data (AUC = 0.76). Ciobanu et al. [14] proposed a fuzzy forests machine learning model which was able to estimate recurrent MDD with an accuracy of 63% in an elderly population using transcriptome data. Liu et al. [15] utilized an elastic net machine learning algorithm to predict MDD using self-reported questionnaires (AUC = 0.78). Finally, a recent study by Arloth et al. [16] reported an integrated machine learning and genome-wide analysis approach to identify regulatory SNPs which are associated with MDD using expression quantitative trait loci (eQTLs) and methylation quantitative trait loci information.

Numerous genome-wide association studies (GWASs) have been performed to identify genetic variants associated with MDD. For instance, Ripke et al. [17] performed a GWAS meta-analysis study of MDD in individuals of European ancestry (9240 MDD cases and 9519 controls) and found no SNPs at genome-wide significance level. In a subsequent GWAS meta-analysis study, Wray et al. [18] detected 44 variants at genome-wide significance level for MDD in individuals of European ancestry (135,458 MDD cases and 344,901 controls). In a recent GWAS meta-analysis study, Howard et al. [19] identified 102 genome-wide significant variants in European populations (246,363 MDD cases and 561,190 controls). In addition, only a handful of studies have reported sex-specific loci for male or female MDD [20]. For instance, Powers et al. [21] suggested that SNP rs6602398 in *IL2RA* was closely related to male MDD in an African American GWAS. Wang et al. [22] showed that SNPs rs619002 and rs644926 in *EHD3* were linked to female MDD in a Chinese population.

In previous studies [4,5], machine learning models were leveraged to estimate antidepressant treatment outcomes with the top-rated key SNPs acquired from a GWAS. Here, we investigated the feasibility of likely loci by performing a GWAS of probable MDD using a sample of 9828 Taiwanese individuals in the Taiwan Biobank. From our data we identified a genome-wide significant association with probable MDD that has not been previously reported: *FBN1* on chromosome 15. In our analysis, we further discovered associations between probable MDD and SNPs in 17 genes, which may also play a potential role as eQTLs. We subsequently combined 17 eQTL SNPs and eight clinical variables to optimally forecast probable MDD using machine learning models, including logistic ridge regression, support vector machine (SVM), C4.5 decision tree, LogitBoost, and random forests. Moreover, we utilized random undersampling [23] and synthetic minority oversampling [24] techniques to cope with imbalanced data at the data level. To our knowledge, no previous studies have investigated predictive algorithms for probable MDD with random undersampling and synthetic minority oversampling techniques. We found that our random forests model with synthetic minority oversampling showed the best performance in predicting probable MDD based on 17 eQTL SNPs and eight clinical variables.

## 2. Materials and Methods

### 2.1. Study Population

Our original study cohort was composed of 10,939 Taiwanese subjects in the Taiwan Biobank [25]. First, we excluded participants who reported a physician diagnosis of the following psychiatric disorders: bipolar disorder, schizophrenia, dementia, and Parkinson’s disease. We also excluded participants who had a score of greater than or equal to 3 on the Anxiety subscale of the Patient Health Questionnaire-4 (PHQ-4) scale [26]. Consequently, we removed 1111 subjects. We then defined the probable MDD and control groups as follows. For the probable MDD group, we included the remaining participants who reported a physician diagnosis of MDD or had a score of greater than or equal to 3 on the Depression subscale of the PHQ-4. For the control group, we included the rest of the participants who neither reported a physician diagnosis of MDD nor had a score of less than 3 on the Depression subscale of the PHQ-4. As a result, there were 9828 subjects for further analysis, with 2457 subjects in the probable MDD group and 7371 subjects in the control group.

Ethical approval for the study was granted by the Institutional Review Board of the Taiwan Biobank before performing the study (approval number: 201506095RINC). The approved informed consent form was signed by each subject. All experiments were achieved by means of proper regulations and guidelines.

As part of the questionnaire, participants were asked if they had had physical activity recently, if they were current alcohol drinkers or ever-smokers, and their education status [27]. Physical activity was defined as a participant having over 30 min of exercise activity each time, over 3 times a week [27]. A current alcohol drinker was defined as currently drinking 150 mL of alcohol per week for more than six months [27]. An ever-smoker was defined as a person who has ever been a cigarette smoker. A participant’s education status included seven categories: no education (illiterate), homeschooling, elementary school, middle school, high school, college, and graduate school.

### 2.2. Genotyping Data and Quality Controls

DNA was extracted from blood samples by employing QIAamp DNA blood kits following the manufacturer’s instructions (Qiagen, Valencia, CA, USA). The quality of the isolated genomic DNA was evaluated with agarose gel electrophoresis, and the quantity was measured by spectrophotometry [28]. SNP genotyping was conducted by using custom Taiwan BioBank chips and an Axiom Genome-Wide Array Plate System (Affymetrix, Santa Clara, CA, USA). The custom Taiwan BioBank chips were created to collect genetic profiles in Taiwanese subjects by utilizing SNPs on the Axiom Genome-Wide CHB 1 Array (Affymetrix, Santa Clara, CA, USA) and the Human Exome BeadChip (Illumina, Inc., San Diego, CA, USA) [29].

In this study, we implemented quality control procedures for subsequent analysis [27,30], including the following quality criteria for SNP exclusion: failure to achieve Hardy–Weinberg equilibrium (with a *p* value less than 1 × 10^−6^), minor allele frequency (MAF) less than 1%, or a genotyping call rate less than 90%. We determined *p* values for Hardy–Weinberg equilibrium, MAFs, and genotyping call rates using PLINK [31]. After conducting the quality control procedures, there were a total of 477,260 SNPs remaining.

### 2.3. Statistical Analysis

The Kruskal–Wallis test was performed to appraise the difference in the means of two continuous variables [7]. We conducted the chi-square test for categorical data [25]. The criterion for significance was set at *p* < 0.05 for all tests. Data are presented as the mean ± standard deviation.

In addition, we performed HaploReg (compbio.mit.edu/HaploReg accessed on 11 April 2021) [32] to measure if there is a functional role as eQTLs for the SNPs in the specific genes.

The Manhattan and quantile-quantile (Q-Q) plots were created using the R package ‘qqman’.

### 2.4. Key eQTL SNPs

For predictive modeling of probable MDD, we first selected top-rated SNPs showing evidence of association with probable MDD at a significant association level of *p* < 1 × 10^−4^ in an odds ratio analysis. We next verified these selected SNPs using HaploReg (compbio.mit.edu/HaploReg accessed on 11 April 2021) [32] to see if these SNPs could be considered as eQTLs. As a result, the final selected SNPs are eQTL SNPs that are associated with probable MDD at a significant association level of *p* < 1 × 10^−4^ in an odds ratio analysis.

### 2.5. An Integrated Machine Learning and Genome-Wide Analysis Approach

**Figure 1:**
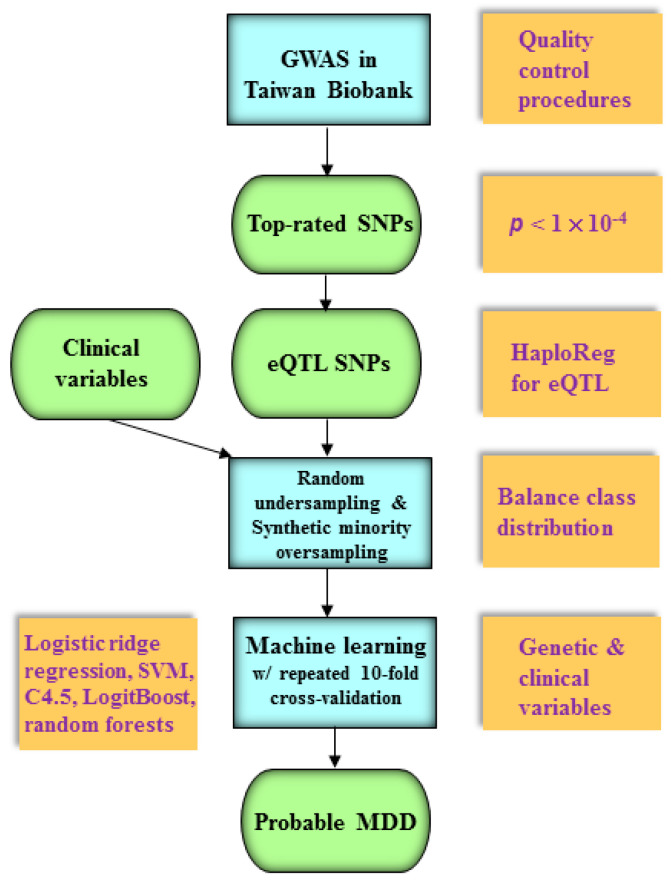
The schematic illustration of an integrated machine learning and genome-wide analysis approach. First, a GWAS is conducted to obtain a set of top-rated SNPs (*p* < 1 × 10^−4^ in an odds ratio analysis) in the Taiwan Biobank. Second, HaploReg (compbio.mit.edu/HaploReg, accessed on 11 April 2021) [32] is used to verify if these top-rated SNPs can be considered as eQTLs. Then, random undersampling and synthetic minority oversampling techniques are employed to eliminate instances in the majority class for balancing class distribution. Finally, five machine learning models (logistic ridge regression, SVM, C4.5 decision tree, LogitBoost, and random forests) with the repeated 10-fold cross-validation approach are utilized to predict probable MDD using eQTL SNPs and clinical variables. eQTLs = expression quantitative trait loci; GWAS = genome-wide association study; MDD = major depressive disorder; SNPs = single nucleotide polymorphisms; SVM = support vector machine.

Figure 1 illustrates the combination of the GWAS and machine learning models with two techniques for imbalanced datasets. We employed five prediction algorithms (see Section 2.6.) for the prediction of probable MDD. We also utilized a random undersampling technique [23] and a synthetic minority oversampling technique [24] for handling imbalanced datasets: the former technique eliminates instances in the majority class to balance class distribution, and the latter is implemented by creating synthetic minority class instances. We combined the machine learning algorithms with these two techniques.

### 2.6. Machine Learning Algorithms for Benchmarking

For the benchmarking task in the present study, we employed five state-of-the-art machine learning algorithms for comparison: logistic ridge regression, SVM, C4.5 decision tree, LogitBoost, and random forests. We performed the analyses for these five machine learning algorithms using WEKA software (which is available from www.cs.waikato.ac.nz/ml/weka/ accessed on 11 April 2021) [33] and a computer with Intel (R) Core (TM) i5-4210U, 4 GB RAM, and Windows 7. The tuning parameters of WEKA were determined at the specific values using a grid search approach [5,34].

The logistic ridge regression model utilizes the ridge estimation technique to enhance the parameter estimates and to reduce the error produced by further predictions [35]. The main method to obtain ridge parameters is the cross-validation approach, so that the model can forecast new observations more accurately. Here, the tuning parameters of WEKA were 10 for the ridge parameter and 100 for the batch size.

The SVM model [36] is a common method for pattern recognition and classification [7]. Given a training set of instance-label pairs, the SVM model leverages a kernel function to map the training vectors into a higher dimensional space [36,37]. In this higher dimensional space, the SVM model then finds a linear separating hyperplane with the maximal margin. In this study, we applied the polynomial kernel with the exponent value of 1.0 [5].

The C4.5 decision tree model builds decision trees top-down and prunes them using the concept of information entropy [33]. First, the tree is constructed by finding the root node (for example, SNPs) that is the most discriminative for differentiating “probable MDD” from “healthy control”. Then, the best single feature test is decided by the information gain and by choosing a feature (for example, SNPs) to split the data into subsets. Here, the tuning parameters of WEKA were 0.1 for the confidence factor and 4 for the minimum number of instances per leaf node [38].

The LogitBoost model is a boosting ensemble model, which incorporates the performance of many weak predictive models to produce a robust predictive model with higher accuracy [5,7]. The base predictive model we utilized was linear regression. Here, the tuning parameters of WEKA were 0.5 for the shrinkage parameter, 100 for the batch size, 3.0 for the Z max threshold, and 10 for the number of iterations.

The random forests model builds a collection of decision trees during training, and then produces the class that is the mode of the classes among the individual trees [39]. Here, the tuning parameters of WEKA for the random forests model were 100 for the batch size and 300 for the number of iterations.

### 2.7. Evaluation of the Predictive Performance

In this study, we applied one of the most common criteria to calculate the performance of the predictive models, utilizing the receiver operating characteristic (ROC) methodology to determine the area under the ROC curve (AUC) [37,38,40]—the higher the AUC, the better the predictive model [38,40]. We also calculated sensitivity (that is, the proportion of correctly predicted probable MDD subjects of all tested probable MDD subjects) and specificity (that is, the proportion of correctly predicted healthy controls of all the tested healthy controls). Additionally, we applied the repeated 10-fold cross-validation approach to assess the generalization of the predictive models [37,38,40]. The whole cohort was randomly divided into ten individual partitions. Then, a training cohort (i.e., nine-tenths of the partitions) and a testing cohort (i.e., the remaining tenth of the partitions) were used to estimate the predictive performance. Next, the previous step was repeated nine more times by choosing different nine-tenths of the partitions for the training cohort and a different tenth of the partitions for the testing cohort. Lastly, the final estimation was evaluated by averaging the aforementioned ten runs.

## 3. Results

### 3.1. The Study Cohort in the Taiwan Biobank

There were 9828 participants in the Taiwanese population, including 2457 subjects with probable MDD and 7371 healthy individuals. As shown in Table 1, age distributions were different between the two groups (*p* = 0.009). The mean age (51.7 ± 10.0 years) of probable MDD subjects was older than that of the healthy controls (51.0 ± 10.5 years). Gender distributions were also different between probable MDD subjects and healthy controls (*p* < 0.001). In addition, education status, marital status, social relationship (assessed by whether or not the subject lived alone), employment status, and status of ever-smoker were significantly different between probable MDD patients and healthy controls (Table 1; all *p* < 0.001). However, there were no significant differences between these two groups for current alcohol drinker (*p* = 0.798) and physical activity (*p* = 0.297) (Table 1).

### 3.2. GWAS of Probable MDD in the Taiwanese Population

**Figure 2:**
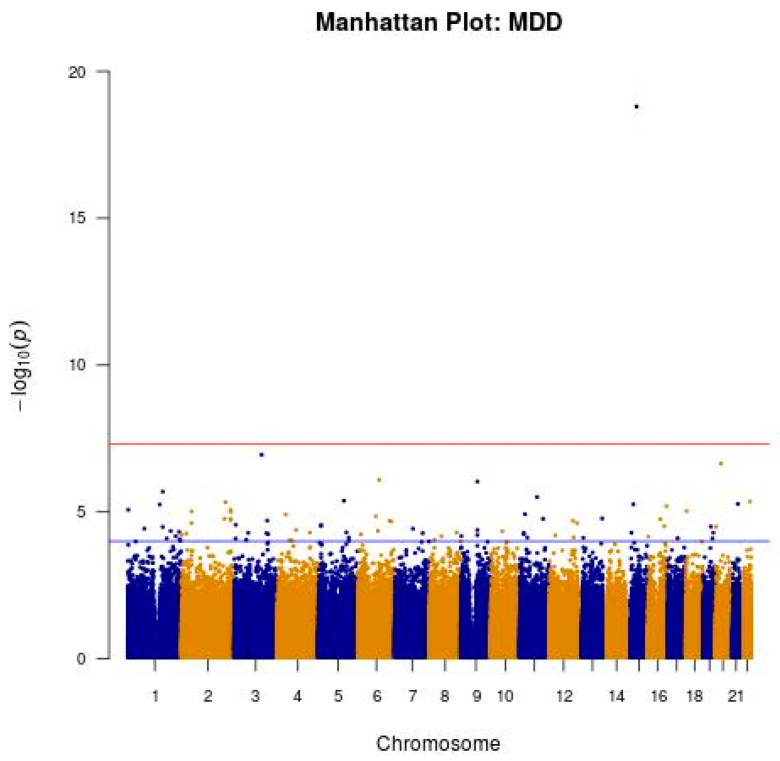
The Manhattan plots of genome-wide association of SNPs with MDD. The Manhattan plots were generated by using the *p* values of SNPs. A single SNP is indicated by a point, where a higher point (higher negative log_10_ *p* value) demonstrates a more significant association. The red horizontal line is displayed as the genome-wide significance level (*p* = 5 × 10^−8^). The blue horizontal line is displayed as the significance level of *p* = 1 × 10^−4^. Hence, points above the blue horizontal line illustrate SNPs with a *p* value of less than 1 × 10^−4^. Y-axis: -log_10_ (*p* value) of each SNP; X-axis: chromosomes demonstrated with blue and orange colors. MDD = major depressive disorder; SNPs = single nucleotide polymorphisms.

We conducted a GWAS of probable MDD in the Taiwan Biobank using a sample of 9828 individuals. Figure 2 illustrates the Manhattan plots of the association *p* values for the SNPs. The test statistics were properly calibrated genome-wide, as illustrated by the Q-Q plot of the association results (Supplementary Figure S1) and a genomic control inflation factor of 0.963.

SNP rs193922209 (*p* = 1.57 × 10^−19^) in the intron region of the *FBN1* gene on chromosome 15 at 15q21.1 is novel, and is associated with probable MDD at the genome-wide significance level (Supplementary Table S1). Supplementary Table S2 shows the genotyping call rate and *p* value for Hardy–Weinberg equilibrium for SNP rs193922209. To our knowledge, this is the first GWAS to discover the genome-wide significance level variant in the *FBN1* gene for probable MDD. We confirmed that these GWAS results are novel for probable MDD using the NHGRI-EBI GWAS Catalog [41].

We further investigated sex-specific SNPs for probable male and female MDD. SNP rs114542799 (*p* = 2.95 × 10^−8^) in the intron region of the *ALDH1L1* gene on chromosome 3 at 3q21.3 is novel and is associated with probable female MDD at the genome-wide significance level (Supplementary Table S3). Supplementary Table S4 shows the genotyping call rate and *p* value for Hardy–Weinberg equilibrium for SNP rs114542799. To our knowledge, this is the first GWAS to discover the genome-wide significance level variant in the *ALDH1L1* gene for probable female MDD. We confirmed that these GWAS results are novel for probable female MDD using the NHGRI-EBI GWAS Catalog [41]. We did not identify sex-specific SNPs for probable male MDD.

### 3.3. Key eQTL SNPs for Probable MDD Identified in the Taiwanese Population

In the next step, we investigated the association between probable MDD and key eQTL SNPs assessed in the GWAS study (see Methods). First, we identified SNPs which reached the significance level of *p* < 1 × 10^−4^ (Figure 2). We then directly verified these significant SNPs to see if they have likely roles as eQTLs. For further investigation in the subsequent analyses, we obtained 17 eQTL SNPs showing likely roles as eQTLs as well as evidence of associations with probable MDD per se (Table 2).

As shown in Table 2, the top-rated 17 eQTL SNPs encompass rs11240075 near *LINC00624-BCL9*, rs3813628 in *TOMM40L* (or *MIR5187*), rs2307424 in *NR1I3*, rs12040314 near *CEP350-QSOX1*, rs1443524 in *LOC105377123*, rs12516830 near *CTNND2-RNU6-679P*, rs11241959 in *FBN2*, rs3734669 in *MCUR1*, rs6558174 in *BIN3*, rs4767012 in *RPH3A*, rs9511242 near *CYCSP33-PARP4*, rs9559849 near *RAB20-NAXD*, rs7403037 in *PWRN1*, rs66649828 in *METRN*, rs7188498 in *LOC101928474*, rs12978607 near *EEF1A1P7-LINC01531*, and rs11083840 in *PTGIR.* For instance, for SNP rs3813628 in the 5′-UTR region of the *TOMM40L* (or *MIR5187*) gene, there was an indication of an association with probable MDD after adjustment of covariates such as age and gender (Table 2; OR = 0.77; 95% CI = 0.69–0.86; *p* = 2.04 × 10^−6^). Supplementary Table S5 shows genotyping call rates and *p* values for Hardy–Weinberg equilibrium for these 17 eQTL SNPs.

Supplementary Table S6 shows the likely roles of the above 17 SNPs as eQTLs. For instance, rs66649828 in *METRN* is involved in regulating expressions of its own gene in various tissues, such as brain cerebellar hemisphere, brain cerebellum, and nerve tibial tissues [42] (Supplementary Table S4). Note that there was no potential mechanism for rs193922209 in *FBN1* or for rs114542799 in *ALDH1L1* as an eQTL.

Supplementary Table S7 summarizes SNPs that were associated with MDD in the previous GWAS reports from the NHGRI-EBI GWAS Catalog [41]. Among these SNPs, rs12516830 in the *CTNND2-RNU6-679P* locus identified in this study is nearby to three SNPs, namely rs6893200, rs2964802, and rs2964802, in the *DAP-CTNND2* locus from a previous GWAS report [43] (Supplementary Table S8). In addition, rs9559849 in the *RAB20-NAXD* locus is nearby to rs4438172 in the *RPL21P107-LINC00567* locus (Supplementary Table S8).

### 3.4. Prediction of Probable MDD with a Random Undersampling Technique

We employed 25 biomarkers, including the aforementioned 17 key eQTL SNPs and eight clinical variables, to build the predictive models for differentiating probable MDD from healthy controls by employing five machine learning algorithms with random undersampling (see Methods). The selected eight clinical variables encompass age, gender, education status, marital status, social relationship (assessed by whether the individual lived alone), employment status, status of ever-smoking (Table 1; all *p* < 0.001), and current alcohol drinking (Table 1; *p* = 0.798). The clinical variable regarding current alcohol drinking was included as alcohol consumption might contribute to any underlying condition of sadness/melancholy in MDD subjects with a comorbid condition of alcoholism [44,45]. The clinical variable regarding physical activity was not included, as this clinical variable was not linked to probable MDD in this study (Table 1; *p* = 0.297).

To evaluate the performance of our approach for predictive models for probable MDD, we compared five state-of-the-art methods, namely logistic ridge regression, SVM, C4.5 decision tree, LogitBoost, and random forests (Table 3). In terms of AUC for probable MDD, the logistic ridge regression model obtained comparable performance as the LogitBoost model (Table 3; AUC = 0.8242 ± 0.0176 and 0.8246 ± 0.0176, respectively). These two models outperformed SVM, C4.5 decision tree, and random forests in terms of AUC (Table 3). Supplementary Figure S2 shows the comparison of ROC plots between logistic ridge regression and the other four benchmarking models. Based on the ROC plots, logistic ridge regression had a similar performance as LogitBoost as the two curves were overlaid (Supplementary Figure S2).

### 3.5. Prediction of Probable MDD with a Synthetic Minority Oversampling Technique

Next, we employed the aforementioned 25 biomarkers to build predictive models for differentiating probable MDD from healthy controls by employing five machine learning algorithms with synthetic minority oversampling (see Methods). Table 4 shows experiment results for predicting probable MDD with synthetic minority oversampling using five state-of-the-art methods: logistic ridge regression, SVM, C4.5 decision tree, LogitBoost, and random forests. In terms of AUC for probable MDD, the random forests model obtained the maximal AUC among the predictive models for probable MDD, where the best AUC was 0.8905 ± 0.0088 (Table 4), outperforming logistic ridge regression, SVM, C4.5 decision tree, and LogitBoost in terms of AUC (Table 4). Supplementary Figure S3 shows the comparison of ROC plots between random forests and the other four benchmarking models.

After comparing all the predictive models (Table 3 and Table 4), the random forests model with synthetic minority oversampling had the highest performance overall. Our analysis indicates that the random forests model is well-suited for predictive models for probable MDD.

## 4. Discussion

To our knowledge, this is the first study to date to explore an integrated machine learning and genome-wide analysis approach for the prediction of probable MDD among Taiwanese individuals in the Taiwan Biobank using eQTL SNPs and clinical biomarkers. Moreover, we observed for the first time that the *FBN1* gene on chromosome 15 is associated with probable MDD at genome-wide significance level in Taiwanese individuals. In addition, we conducted the first study to foresee plausible biomarkers, including 17 eQTL SNPs and eight clinical variables, in influencing probable MDD. The findings indicate that the random forests model with synthetic minority oversampling surpassed logistic ridge regression, SVM, C4.5 decision tree, and LogitBoost in terms of AUC for forecasting probable MDD.

Intriguingly, the present study is the first to raise the possibility that *FBN1* is significantly associated with probable MDD. The significant association between *FBN1* and probable MDD reached the genome-wide significance level. We confirmed that these GWAS results are novel for probable MDD using the NHGRI-EBI GWAS Catalog [41]. The *FBN1* gene, located on chromosome 15 at 15q21.1, primarily encodes the fibrillin protein, which is an essential component of elastic fibers in connective tissue throughout the body [46]. The *FBN1* gene is a strong candidate for probable MDD, as this gene has been previously implicated in mental disorders. For example, Djurovic et al. [47] reported that *FBN1* was moderately associated with bipolar disorder in a Norwegian GWAS, where bipolar disorder is a mental illness that causes severe high (mania) and low (depression) moods.

We pinpointed 17 eQTL SNPs in or near 17 loci which possess potential eQTL mechanisms, as well as associations with probable MDD, where the 17 loci are *LINC00624-BCL9*, *TOMM40L* (or *MIR5187*), *NR1I3*, *CEP350-QSOX1*, *LOC105377123*, *CTNND2-RNU6-679P*, *FBN2*, *MCUR1*, *BIN3*, *RPH3A*, *CYCSP33-PARP4*, *RAB20-NAXD*, *PWRN1*, *METRN*, *LOC101928474*, *EEF1A1P7-LINC01531*, and *PTGIR*. It has been indicated that integrating regulatory information (such as eQTLs) enhances the power to identify functional SNPs that may play a key role in the etiology of the disease [16]. In agreement with our results, Li et al. [48] suggested that the *BCL9* gene confers risk of MDD in the Chinese Han population. Nivard et al. [49] also reported evidence for an association between *CTNND2* and MDD using GWAS data from the Psychiatric Genomics Consortium. In addition, Dunn et al. [50] showed that SNP rs4652467 near *CEP350* interacts with stressful life events to influence MDD in an African American GWAS. In contrast, to the best of our knowledge, no previous studies have shown that the other novel loci may contribute to MDD.

Another intriguing finding was that seven clinical variables were discovered to be substantially linked to probable MDD: age, gender, education status, marital status, social relationship (assessed by whether the individual lived alone), employment status, and status of ever-smoking. In line with our study, it has been previously suggested that marital status (or relationship status) is associated with the development and severity of MDD [51]. A link between MDD and unemployment was reported in an international study [52] and a Finland study [53]. It has also been shown that MDD and smoking are highly correlated [54,55]. Murcia et al. [56] observed that educational inequalities contributed to MDD in a French national study. Finally, it has been indicated that social relationship [57], age [58], and gender [59] are correlated with MDD.

The present study is the first to raise the possibility that *ALDH1L1* is significantly associated with probable female MDD. Furthermore, the significant association between *ALDH1L1* and probable female MDD reached the genome-wide significance level. We confirmed that these GWAS results are novel for probable female MDD using the NHGRI-EBI GWAS Catalog [41]. The *ALDH1L1* gene, located on chromosome 3 at 3q21.3, is an astrocyte marker, where astrocytes are glial cells in the central nervous system and linked to various brain functions and MDD [60]. The *ALDH1L1* gene is a strong candidate for probable MDD, as this gene has been previously implicated in MDD and suicide [61,62].

This study had some limitations. First, due to a lack of time series data, we were unable to perform time series-based deep learning models such as one-dimensional convolutional neural network and long short-term memory models [63]. Similarly, due to a lack of time series data, we were unable to divide training and testing datasets separately according to a timeline, for example, patients before 2015 for training and the patients beyond 2015 for testing in the cross-validation procedure. It is hypothesized that using the whole dataset without a certain timeframe in the cross-validation procedure might disguise any cohort-specific effects on how MDD might manifest in the patients as time passed [64,65,66]. On the other hand, the main advantage of machine learning methods over deep learning approaches is that extensive computing resources (i.e., general-purpose computing on graphics processing units), which are normally utilized in deep learning algorithms, are in general not required for implementing machine learning models [67,68].

## 5. Conclusions

In conclusion, we propose an integrated machine learning and genome-wide analysis approach to identify eQTL SNPs and predict probable MDD in the Taiwan Biobank. The present results suggest that our random forests model with synthetic minority oversampling may present a feasible way to create predictive algorithms for forecasting MDD with clinically meaningful accuracy. The analysis of the present study might be generalized for machine learning studies of personalized medicine in predicting disease status and treatment response for individuals. Moreover, the results can be utilized to build bioinformatics tools for personalized medicine within the next few years. Finally, it is crucial to further investigate the role of our proposed predictive framework by using various independent samples of replication studies.

## Figures and Tables

**Table 1:** Demographic and clinical characteristics of study subjects.

Characteristic	Overall	Probable MDD	Control	*p*
No. of subjects, *n*	9828	2457	7371	
Mean age ± SD, years	51.2 ± 10.4	51.7 ± 10.0	51.0 ± 10.5	0.009
Male (%)	25.2%	19.8%	26.8%	<0.001
Education ^1^, *n* (seven categories)	12/7/619/835/ 3133/4402/811	3/2/149/247/ 857/1022/174	9/5/470/588 2276/3380/637	<0.001
Married, *n*	7006	1486	5520	<0.001
Lived alone, *n*	943	348	595	<0.001
Currently employed, *n*	4719	252	4467	<0.001
Current alcohol drinker, *n*	433	106	327	0.798
Ever-smoker, *n*	2312	701	1611	<0.001
Any physical activity, *n*	4413	1081	3332	0.297

MDD = major depressive disorder; SD = standard deviation. Data are presented as mean ± standard deviation. ^1^ Education status is defined as the following seven categories: no education (illiterate), homeschooling, elementary school, middle school, high school, college, and graduate school.

**Table 2:** Odds ratio analysis with odds ratios after adjustment for covariates between probable MDD and 17 eQTL SNPs.

CHR	Gene	SNP	A1	A2	Region	MAF	OR	95% CI	*p*
1	*LINC00624-BCL9*	rs11240075	T	C	intergenic	0.247	1.68	(1.34–2.10)	5.60 × 10^−6^
1	*TOMM40L, MIR5187*	rs3813628	A	C	5′-UTR	0.465	0.77	(0.69–0.86)	2.04 × 10^−6^
1	*NR1I3*	rs2307424	G	A	synonymous	0.476	0.80	(0.72–0.89)	3.26 × 10^−5^
1	*CEP350-QSOX1*	rs12040314	G	A	intergenic	0.247	0.83	(0.76–0.91)	8.03 × 10^−5^
3	*LOC105377123*	rs1443524	G	A	intronic	0.326	1.39	(1.18–1.63)	5.18 × 10^−5^
5	*CTNND2-RNU6-679P*	rs12516830	T	C	intergenic	0.250	0.82	(0.75–0.90)	2.79 × 10^−5^
5	*FBN2*	rs11241959	G	A	intronic	0.180	0.82	(0.74–0.90)	4.94 × 10^−5^
6	*MCUR1*	rs3734669	T	G	3′-UTR	0.453	0.80	(0.72–0.89)	5.87 × 10^−5^
8	*BIN3*	rs6558174	A	G	intronic	0.270	1.48	(1.22–1.81)	9.11 × 10^−5^
12	*RPH3A*	rs4767012	G	A	intronic	0.275	0.72	(0.61–0.85)	7.34 × 10^−5^
13	*CYCSP33-PARP4*	rs9511242	A	G	intergenic	0.349	0.83	(0.75–0.91)	7.61 × 10^−5^
13	*RAB20-NAXD*	rs9559849	A	G	intergenic	0.470	1.29	(1.15–1.44)	1.68 × 10^−5^
15	*PWRN1*	rs7403037	G	T	intronic	0.160	0.56	(0.43–0.74)	5.13 × 10^−5^
16	*METRN*	rs66649828	A	G	intronic	0.405	1.21	(1.10–1.33)	6.98 × 10^−5^
16	*LOC101928474*	rs7188498	A	G	intronic	0.183	0.60	(0.48–0.75)	6.40 × 10^−6^
19	*EEF1A1P7-LINC01531*	rs12978607	A	C	intergenic	0.490	1.24	(1.12–1.38)	3.19 × 10^−5^
19	*PTGIR*	rs11083840	G	T	intronic	0.416	0.79	(0.70–0.88)	5.12 × 10^−5^

A1 = minor allele, A2 = major allele, CHR = chromosome, CI = confidence interval, MAF = minor allele frequency, MDD = major depressive disorder, OR = odds ratio. Analysis was obtained after adjustment for covariates including age and gender.

**Table 3:** The results of repeated 10-fold cross-validation experiments with a random undersampling technique for differentiating MDD patients from healthy individuals, using logistic ridge regression, SVM, C4.5 decision tree, LogitBoost, and random forests with biomarkers including eight clinical variables and 17 SNPs.

Algorithm	AUC	Sensitivity	Specificity
Logistic ridge regression	0.8242 ± 0.0176	0.7618 ± 0.0177	0.7618 ± 0.0177
SVM	0.7576 ± 0.0185	0.7576 ± 0.0185	0.7576 ± 0.0185
C4.5 decision tree	0.7586 ± 0.0203	0.7571 ± 0.0187	0.7571 ± 0.0187
LogitBoost	0.8246 ± 0.0176	0.7619 ± 0.0171	0.7619 ± 0.0171
Random forests	0.8179 ± 0.0185	0.7588 ± 0.0186	0.7588 ± 0.0186

AUC = the area under the receiver operating characteristic curve; MDD = Major Depressive Disorder; SVM = support vector machine.

**Table 4:** The results of repeated 10-fold cross-validation experiments with a synthetic minority oversampling technique for differentiating MDD patients from healthy individuals, using logistic ridge regression, SVM, C4.5 decision tree, LogitBoost, and random forests with biomarkers such as eight clinical variables and 17 SNPs.

Algorithm	AUC	Sensitivity	Specificity
Logistic ridge regression	0.8557 ± 0.0100	0.7772 ± 0.0126	0.7674 ± 0.0146
SVM	0.7681 ± 0.0061	0.7592 ± 0.0082	0.7771 ± 0.0060
C4.5 decision tree	0.8370 ± 0.0110	0.7845 ± 0.0104	0.7636 ± 0.0124
LogitBoost	0.8559 ± 0.0100	0.7778 ± 0.0127	0.7688 ± 0.0145
Random forests	0.8905 ± 0.0088	0.8072 ± 0.0102	0.7860 ± 0.0124

AUC = the area under the receiver operating characteristic curve; MDD = Major Depressive Disorder; SVM = support vector machine.

## Data Availability

The data that support the findings of this study are available from the Taiwan Biobank. To apply for access to these third-party data, please contact the Taiwan Biobank at biobank@gate.sinica.edu.tw.

## Supplementary Materials

The following are available online at https://www.mdpi.com/article/10.3390/jpm11070597/s1, Figure S1: The quantile-quantile (Q-Q) plot of probable major depressive disorder (MDD) results, Figure S2: Plots of receiver operating characteristic (ROC) curves using random undersampling, Figure S3: Plots of receiver operating characteristic (ROC) curves using synthetic minority oversampling, Table S1: Odds ratio analysis with odds ratios after adjustment for covariates between MDD and rs193922209 in *FBN1*, Table S2: Genotyping results for rs193922209 in *FBN1*, Table S3: Odds ratio analysis with odds ratios after adjustment for covariates between female MDD and rs114542799 in *ALDH1L1*, Table S4: Genotyping results for rs114542799 in *ALDH1L1*, Table S5: Genotyping results for 17 eQTL SNPs, Table S6: The eQTL roles of the 17 eQTL SNPs, Table S7: Summary of SNPs associated with major depressive disorder in previous reports from the NHGRI-EBI GWAS Catalog, Table S8: Nearby SNPs between the Taiwan Biobank and previous reports from the NHGRI-EBI GWAS Catalog.
