# Supplementary material for: Prediction of Probable Major Depressive Disorder in the Taiwan Biobank: An Integrated Machine Learning and Genome-Wide Analysis Approach
Source: J Pers Med. 2021 Jun 24;11(7):597. doi: 10.3390/jpm11070597 (PMC8308113; doi:10.3390/jpm11070597)
Supplement: Supplementary file 1 [file jpm-11-00597-s001.zip › jpm-1229212-Supplementary/Supp-Table-S1.pdf]

Table S1. Odds ratio analysis with odds ratios after adjustment for covariates between MDD and rs193922209 in *FBN1* .

CHR	Gene	SNP	A1	A2	Region	MAF	OR	95% CI	P
15	*FBN1*	rs193922209	G	C	intronic	0.040	3.06	(2.40 – 3.90)	1.57×10^{-19}

A1 = minor allele, A2 = major allele, CHR = chromosome, CI = confidence interval, MAF = minor allele frequency, MDD = major depressive disorder, OR = odds ratio.

Analysis was obtained after adjustment for covariates including age and gender.
