# Supplementary material for: Prediction of Probable Major Depressive Disorder in the Taiwan Biobank: An Integrated Machine Learning and Genome-Wide Analysis Approach
Source: J Pers Med. 2021 Jun 24;11(7):597. doi: 10.3390/jpm11070597 (PMC8308113; doi:10.3390/jpm11070597)
Supplement: Supplementary file 1 [file jpm-11-00597-s001.zip › jpm-1229212-Supplementary/Supp-Table-S2.pdf]

Table S2. Genotyping results for rs193922209 in *FBN1* .

| CHR | Gene | SNP | A1 | A2 | MAF | Genotyping call rate | P (HWE) |
|---|---|---|---|---|---|---|---|
| 15 | *FBN1* | rs193922209 | G | C | 0.040 | 0.998 | $3.65 \times 10^{-6}$ |

A1 = minor allele, A2 = major allele, Chr = chromosome, HWE = Hardy–Weinberg equilibrium, MAF = minor allele frequency.
