# Supplementary material for: Prediction of Probable Major Depressive Disorder in the Taiwan Biobank: An Integrated Machine Learning and Genome-Wide Analysis Approach
Source: J Pers Med. 2021 Jun 24;11(7):597. doi: 10.3390/jpm11070597 (PMC8308113; doi:10.3390/jpm11070597)
Supplement: Supplementary file 1 [file jpm-11-00597-s001.zip › jpm-1229212-Supplementary/Supp-Table-S3.pdf]

Table S3. Odds ratio analysis with odds ratios after adjustment for covariates between female MDD and rs114542799 in *ALDH1L1* .

CHR	Gene	SNP	A1	A2	Region	MAF	OR	95% CI	P
3	*ALDH1L1*	rs114542799	A	G	intronic	0.011	0.40	(0.29 – 0.56)	2.95×10^{-8}

A1 = minor allele, A2 = major allele, CHR = chromosome, CI = confidence interval, MAF = minor allele frequency, MDD = major depressive disorder, OR = odds ratio.
Analysis was obtained after adjustment for covariates including age.
