# Supplementary material for: Prediction of Probable Major Depressive Disorder in the Taiwan Biobank: An Integrated Machine Learning and Genome-Wide Analysis Approach
Source: J Pers Med. 2021 Jun 24;11(7):597. doi: 10.3390/jpm11070597 (PMC8308113; doi:10.3390/jpm11070597)
Supplement: Supplementary file 1 [file jpm-11-00597-s001.zip › jpm-1229212-Supplementary/Supp-Table-S4.pdf]

Table S4. Genotyping results for rs114542799 in *ALDH1L1* .

CHR	Gene	SNP	A1	A2	MAF	Genotyping call rate	P (HWE)
3	*ALDH1L1*	rs114542799	A	G	0.011	0.999	1.00

A1 = minor allele, A2 = major allele, Chr = chromosome, HWE = Hardy–Weinberg equilibrium, MAF = minor allele frequency.
