# Supplementary material for: Prediction of Probable Major Depressive Disorder in the Taiwan Biobank: An Integrated Machine Learning and Genome-Wide Analysis Approach
Source: J Pers Med. 2021 Jun 24;11(7):597. doi: 10.3390/jpm11070597 (PMC8308113; doi:10.3390/jpm11070597)
Supplement: Supplementary file 1 [file jpm-11-00597-s001.zip › jpm-1229212-Supplementary/Supp-Table-S5.pdf]

Table S5. Genotyping results for 17 eQTL SNPs.

CHR	Gene	SNP	A1	A2	MAF	Genotyping call rate	P (HWE)
1	*LINC00624-BCL9*	rs11240075	T	C	0.247	0.999	0.166
1	*TOMM40L, MIR5187*	rs3813628	A	C	0.465	0.997	0.626
1	*NR1I3*	rs2307424	G	A	0.476	0.996	0.792
1	*CEP350-QSOX1*	rs12040314	G	A	0.247	0.998	0.290
3	*LOC105377123*	rs1443524	G	A	0.326	0.997	0.334
5	*CTNND2-RNU6-679P*	rs12516830	T	C	0.250	0.998	0.187
5	*FBN2*	rs11241959	G	A	0.180	0.998	0.182
6	*MCUR1*	rs3734669	T	G	0.453	0.998	0.514
8	*BIN3*	rs6558174	A	G	0.270	0.995	0.124
12	*RPH3A*	rs4767012	G	A	0.275	0.999	0.494
13	*CYCSP33- PARP4*	rs9511242	A	G	0.349	0.997	0.842
13	*RAB20-NAXD*	rs9559849	A	G	0.470	0.997	0.670
15	*PWRN1*	rs7403037	G	T	0.160	0.999	0.033
16	*METRN*	rs66649828	A	G	0.405	0.997	0.933
16	*LOC101928474*	rs7188498	A	G	0.183	0.999	0.028
19	*EEF1A1P7- LINC01531*	rs12978607	A	C	0.490	0.998	0.455
19	*PTGIR*	rs11083840	G	T	0.416	0.998	0.678

A1 = minor allele, A2 = major allele, Chr = chromosome, HWE = Hardy–Weinberg equilibrium, MAF = minor allele frequency.
