# Supplementary material for: Prediction of Probable Major Depressive Disorder in the Taiwan Biobank: An Integrated Machine Learning and Genome-Wide Analysis Approach
Source: J Pers Med. 2021 Jun 24;11(7):597. doi: 10.3390/jpm11070597 (PMC8308113; doi:10.3390/jpm11070597)
Supplement: Supplementary file 1 [file jpm-11-00597-s001.zip › jpm-1229212-Supplementary/Supp-Table-S6.pdf]

Table S6. The eQTL roles of the 17 eQTL SNPs.

CHR	Gene	SNP	Tissue (for eQTL study)	Reference (PMID for eQTL study)
1	*LINC00624-BCL9*	rs11240075	Lymphoblastoid EUR Exonlevel	24037378
1	*TOMM40L, MIR5187*	rs3813628	Muscle Skeletal, Skin Sun Exposed Lower Leg, Whole Blood	25954001, 24013639
1	*NR1I3*	rs2307424	Muscle Skeletal , Whole Blood	25954001, 24013639
1	*CEP350-QSOX1*	rs12040314	Testis, Whole Blood	25954001, 24013639
3	*LOC105377123*	rs1443524	Stomach	25954001
5	*CTNND2-RNU6-679P*	rs12516830	Nerve Tibia, Thyroid	25954001
5	*FBN2*	rs11241959	Adrenal Gland, Artery Tibial, Colon Sigmoid, Esophagus Muscularis, Lung, Thyroid, Whole Blood	25954001, 24013639
6	*MCUR1*	rs3734669	Skin Sun Exposed Lower Leg, Thyroid, Lymphoblastoid EUR Exonlevel	25954001, 24037378
8	*BIN3*	rs6558174	Whole Blood	24013639
12	*RPH3A*	rs4767012	Muscle Skeletal, Whole Blood	25954001, 24013639
13	*CYCSP33- PARP4*	rs9511242	Cells Transformed Fibroblasts, Esophagus Muscularis, Lymphoblastoid EUR Exonlevel	25954001, 24037378
13	*RAB20-NAXD*	rs9559849	Whole Blood	24013639
15	*PWRN1*	rs7403037	Brain Caudate Basal Ganglia, Brain Putamen Basal Ganglia, Thyroid	25954001
16	*METRN*	rs66649828	Brain Cerebellar Hemisphere, Brain Cerebellum, Adipose Subcutaneous, Adipose Visceral Omentum, Artery Aorta, Artery Tibial, Cells Transformed fibroblasts, Colon Sigmoid, Colon Transverse, Esophagus Mucosa, Esophagus Muscularis, Heart Left Ventricle, Lung, Nerve Tibial, Skin Sun Exposed Lower Leg, Testis, Thyroid, Whole Blood, Lymphoblastoid EUR Exonlevel	25954001, 24037378
16	*LOC101928474*	rs7188498	Whole Blood	24013639
19	*EEF1A1P7- LINC01531*	rs12978607	Whole Blood	24013639
19	*PTGIR*	rs11083840	Lymphoblastoid EUR Genelevel, Whole Blood	24037378, 24013639

CHR = chromosome. eQTL = expression quantitative trait locus, PMID = PubMed ID.
The eQTL information is obtained using HaploReg (http://compbio.mit.edu/HaploReg).
