# Supplementary material for: Prediction of Probable Major Depressive Disorder in the Taiwan Biobank: An Integrated Machine Learning and Genome-Wide Analysis Approach
Source: J Pers Med. 2021 Jun 24;11(7):597. doi: 10.3390/jpm11070597 (PMC8308113; doi:10.3390/jpm11070597)
Supplement: Supplementary file 1 [file jpm-11-00597-s001.zip › jpm-1229212-Supplementary/Supp-Table-S7.pdf]

Table S7. Summary of SNPs associated with major depressive disorder in previous reports from the NHGRI-EBI GWAS Catalog.

Chr	Gene	SNP	Risk Allele Frequency	Beta	95% CI	P	First Author
1	*LINC01128*	rs2977608	0.259	1.07	[1.04–1.1]	8.00×10^{-6}	Coleman JRI
1	*LINC02782 - AL139823.1*	rs912988	0.363	0.18	[0.1–0.26] unit increase	6.00×10^{-6}	GENDEP Investigators
1	*CAMTA1*	rs7512599	0.708	0.00	[0.002–0.0044] unit increase	5.00×10^{-7}	Howard DM
1	*RERE*	rs301795	NR	5.77	z score decrease	8.00×10^{-9}	Nagel M
1	*RERE*	rs301806	0.552	0.03	[0.015–0.039] unit decrease	2.00×10^{-9}	Hyde CL
1	*RERE-AS1, RERE*	rs301807	NR	6.41	z score increase	1.00×10^{-10}	Nagel M
1	*RERE, RERE-AS1*	rs301799	0.431	1.03	[1.02–1.03]	4.00×10^{-31}	Howard DM
1	*RERE*	rs159963	0.440	1.03	[1.02–1.04]	3.00×10^{-8}	Wray NR
1	*RERE*	rs6695867	NR	5.86	z score decrease	5.00×10^{-9}	Nagel M
1	*KLHDC7A - DYNLL1P3*	rs4501813	0.940	1.07	[1.04–1.1]	8.00×10^{-6}	Coleman JRI
1	*AL391650.2 - TRIM63*	rs114098943	0.958	1.07	[1.04–1.1]	4.00×10^{-6}	Coleman JRI
1	*AL590434.1 - ZMYM4*	rs111796550	0.037	1.07	[1.04–1.1]	8.00×10^{-6}	Coleman JRI
1	*FTLP18 - AL596257.1*	rs218987	0.305	0.01	[0.0047–0.0101] unit increase	1.00×10^{-7}	Howard DM
1	*AL596257.1 - AC117945.2*	rs1002656	0.297	1.02	[1.02–1.03]	5.00×10^{-19}	Howard DM
1	*AL596257.1 - AC117945.2*	rs4653218	0.579	0.01	[0.0039–0.0087] unit decrease	1.00×10^{-7}	Howard DM
1	*AL353604.1*	rs1466887	0.449	1.01	[1.01–1.02]	1.00×10^{-8}	Howard DM
1	*SKINT1L*	rs41287894	NR	0.45	[0.25–0.65] unit increase	3.00×10^{-6}	Hall LS
1	*ELAVL4*	rs11579246	0.907	1.03	[1.025–1.04]	9.00×10^{-18}	Howard DM
1	*NRDC*	rs1890946	0.533	1.02	[1.02–1.02]	9.00×10^{-19}	Howard DM
1	*SGIP1*	rs10127497	0.138	0.01	[0.0064–0.013] unit increase	1.00×10^{-8}	Howard DM
1	*SGIP1*	rs10789214	0.566	1.01	[1.009–1.018]	4.00×10^{-10}	Howard DM
1	*SGIP1*	rs6679379	0.288	0.01	[0.0047–0.0097] unit increase	3.00×10^{-8}	Howard DM
1	*AL513166.1*	rs2568958	0.616	1.03	[1.03–1.039]	4.00×10^{-52}	Howard DM
1	*AL513166.1*	rs11209948	0.639	0.04	[0.024–0.047] unit increase	8.00×10^{-11}	Hyde CL
1	*AL513166.1*	rs1432639	0.630	1.04	[1.03–1.05]	5.00×10^{-15}	Wray NR
1	*AL513166.1*	rs6687024	0.623	1.03	[NR]	4.00×10^{-8}	Li X
1	*AL513166.1*	rs6699744	0.612	1.04	[1.03–1.05]	7.00×10^{-14}	Cai N
1	*AL513166.1*	rs6699744	0.612	0.01	[0.0065–0.0113] unit increase	2.00×10^{-13}	Howard DM
1	*AL513166.1*	rs10789340	0.624	0.01	[0.0062–0.011] unit increase	2.00×10^{-12}	Howard DM
1	*LINC02797, AL583808.1*	rs2422320	0.430			2.00×10^{-11}	Amare AT
1	*AL583808.1, LINC02797*	rs2422321	0.441	0.03	[0.019–0.043] unit increase	3.00×10^{-8}	Hyde CL
1	*AL583808.1, LINC02797*	rs12129942	0.515	0.01	[0.0037–0.0085] unit increase	2.00×10^{-7}	Howard DM
1	*AL732618.1 - KRT8P21*	rs11210111	0.462	0.01	[0.004–0.0088] unit decrease	5.00×10^{-8}	Howard DM
1	*RN7SKP19 - LINC01360*	rs6424532	0.486	0.01	[0.0041–0.0089] unit increase	4.00×10^{-8}	Howard DM
1	*RN7SKP19 - LINC01360*	rs10890020	0.484	1.03	[1.02–1.03]	3.00×10^{-34}	Howard DM
1	*RN7SKP19 - LINC01360*	rs12129573	0.370	1.04	[1.03–1.05]	4.00×10^{-12}	Wray NR
1	*LINC01360 - LINC02238*	rs10890045	0.704	0.01	[0.0039–0.0089] unit decrease	7.00×10^{-7}	Howard DM
1	*HMGB1P18 - AL606519.1*	rs12065553	0.280	0.03	[0.017–0.045] unit increase	1.00×10^{-8}	Hyde CL
1	*HMGB1P18 - AL606519.1*	rs12073845	NR	5.52	z score decrease	3.00×10^{-8}	Nagel M
1	*HMGB1P18 - AL606519.1*	rs2389016	0.280	1.03	[1.02–1.04]	1.00×10^{-8}	Wray NR
1	*HMGB1P18 - AL606519.1*	rs113188507	0.284	1.02	[1.019–1.029]	3.00×10^{-21}	Howard DM
1	*AC098656.1 - AL627316.1*	rs4261101	0.630	1.03	[1.02–1.04]	1.00×10^{-8}	Wray NR
1	*AC098656.1 - AL627316.1*	rs4356032	0.375	0.03	[0.017–0.041] unit decrease	3.00×10^{-6}	Hyde CL
1	*BCAR3*	rs62001028	NR			5.00×10^{-7}	Wong ML
1	*AC114485.1*	rs116071213	0.040	1.07	[1.04–1.1]	4.00×10^{-6}	Coleman JRI
1	*AKNAD1, CLCC1*	rs114508355	NR	0.46	[0.26–0.66] unit decrease	9.00×10^{-6}	Hall LS
1	*ATXN7L2*	rs12049330	0.140	1.44		6.00×10^{-6}	Shi J
1	*GNAI3*	rs6537837	0.170	1.43		1.00×10^{-6}	Shi J
1	*SELENBP1 - PSMB4*	rs4971040	0.841	0.01	[0.0053–0.0115] unit increase	2.00×10^{-7}	Howard DM
1	*PKLR*	rs115736167	0.020	0.46	[0.28–0.64] unit decrease	2.00×10^{-7}	Hall LS
1	*AL135926.1*	rs1765516	0.543	0.01	[0.0035–0.0083] unit decrease	5.00×10^{-7}	Howard DM
1	*RPS29P5 - COP1*	rs10913112	0.623	1.02	[1.02–1.03]	2.00×10^{-20}	Howard DM
1	*ASTN1*	rs7548151	0.084	0.01	[0.0084–0.0166] unit increase	4.00×10^{-9}	Howard DM
1	*ASTN1*	rs6697602	0.083	1.06	[1.04–1.07]	6.00×10^{-9}	Cai N
1	*AL136114.1, LINC01645*	rs736840	0.095	0.05	[0.03–0.069] unit increase	8.00×10^{-8}	Hyde CL
1	*AL136114.1*	rs72710803	0.088	1.02	[1.02–1.03]	1.00×10^{-10}	Howard DM
1	*AL136114.1*	rs568799986	0.087	0.01	[0.0068–0.015] unit increase	2.00×10^{-7}	Howard DM
1	*CACNA1E*	rs169235	0.247	1.02	[1.01–1.02]	2.00×10^{-11}	Howard DM
1	*ERVMER61-1*	rs1496089	0.736	1.07	[1.04–1.1]	9.00×10^{-6}	Coleman JRI
1	*AL359976.1 - BRINP3*	rs1935881	0.643	0.03	[0.017–0.04] unit decrease	1.00×10^{-6}	Hyde CL
1	*AL136456.1*	rs606149	0.460	1.25	[NR]	3.00×10^{-6}	Lewis CM
1	*CRB1*	rs2759663	0.219	0.01	[0.0048–0.0102] unit decrease	1.00×10^{-7}	Howard DM
1	*DENND1B*	rs12118513	0.215	0.01	[0.0049–0.0103] unit decrease	1.00×10^{-7}	Howard DM
1	*DENND1B*	rs16841842	0.783	0.03	[0.018–0.046] unit increase	5.00×10^{-7}	Hyde CL
1	*DENND1B*	rs17641524	0.209	0.01	[0.0048–0.0102] unit decrease	2.00×10^{-7}	Howard DM
1	*DENND1B*	rs17641524	0.791	1.02	[1.02–1.03]	8.00×10^{-20}	Howard DM

Chr	Gene	SNP	Risk Allele Frequency	Beta	95% CI	P	First Author
1	*DENND1B - C1orf53*	rs9427672	0.760	1.03	[1.02–1.04]	3.00×10^{-8}	Wray NR
1	*LPGAT1*	rs7526682	0.130	5.54	z score increase	3.00×10^{-8}	Peterson RE
1	*ESRRG*	rs2377360	0.263	1.60	[1.39–1.81]	9.00×10^{-6}	GENDEP Investigators
1	*NVL - CNIH4*	rs11579964	0.840	1.19	[NR]	4.00×10^{-6}	Wray NR
1	*STUM - ITPKB*	rs11577545	0.210	5.54	z score increase	3.00×10^{-8}	Peterson RE
1	*BTF3P9 - AL451054.2*	rs12410462	0.122	1.26	[1.16–1.36]	4.00×10^{-7}	GENDEP Investigators
1	*AL353052.1 - BX323014.1*	rs6666516	0.492	1.07	[1.04–1.1]	6.00×10^{-6}	Coleman JRI
1	*AL359764.3, AL359764.2*	rs4660091	0.662	0.03	[0.017–0.04] unit decrease	3.00×10^{-6}	Hyde CL
1	*OR2T12*	rs41310573	NR			4.00×10^{-20}	Wong ML
2	*MIR7515HG - LINC00487*	rs1841556	0.635	1.07	[1.04–1.1]	9.00×10^{-6}	Coleman JRI
2	*AC073062.1 - LINC00276*	rs144356690	0.008	1.07	[1.04–1.1]	5.00×10^{-6}	Coleman JRI
2	*NBAS*	rs10929355	0.456	0.01	[0.005–0.01] unit decrease	6.00×10^{-9}	Howard DM
2	*LAPTM4A-DT - AC098828.1*	rs7565124				2.00×10^{-6}	Aragam N
2	*AC096570.1*	rs11124319	NR	5.62	z score decrease	2.00×10^{-8}	Nagel M
2	*AC096570.1*	rs12052908	0.468	1.02	[1.02–1.03]	4.00×10^{-22}	Howard DM
2	*AC018467.1*	rs1449984	0.720	0.15	[NR] unit decrease	7.00×10^{-6}	Terracciano A
2	*TOGARAM2*	rs882632	0.290	1.34		2.00×10^{-6}	Shi J
2	*SLC8A1-AS1*	rs13011737	NR	0.10	[0.061–0.139] unit increase	1.00×10^{-6}	Hall LS
2	*RTN4*	rs3806572	0.292	0.01	[0.0043–0.0097] unit decrease	8.00×10^{-7}	Howard DM
2	*RTN4 - CLHC1*	rs7568777	0.186	1.08	[1.04–1.11]	1.00×10^{-6}	Coleman JRI
2	*AC073875.1 - ACTG1P22*	rs10175979	0.651	0.01	[0.0037–0.0085] unit increase	8.00×10^{-7}	Howard DM
2	*ACTG1P22*	rs11682175	0.480	1.03	[1.02–1.04]	5.00×10^{-9}	Wray NR
2	*ACTG1P22*	rs11688767	NR	5.53	z score decrease	3.00×10^{-8}	Nagel M
2	*ACTG1P22 - VRK2*	rs1568452	0.385	1.03	[1.025–1.034]	3.00×10^{-40}	Howard DM
2	*ACTG1P22 - VRK2*	rs2717046	0.367	1.03	[NR]	1.00×10^{-8}	Li X
2	*VRK2*	rs12713370	NR	6.04	z score decrease	2.00×10^{-9}	Nagel M
2	*VRK2*	rs1518395	0.613	0.03	[0.02–0.044] unit increase	4.00×10^{-12}	Hyde CL
2	*VRK2*	rs848293	NR	5.64	z score decrease	2.00×10^{-8}	Nagel M
2	*AC007422.1 - LINC01812*	rs724568	0.360	1.17	[NR]	5.00×10^{-6}	Shyn SI
2	*DNAH6*	rs12464040	NR	0.15	[0.091–0.209] unit increase	2.00×10^{-6}	Hall LS
2	*RNF103-CHMP3, AC015971.1*	rs7585722	0.154	1.02	[1.02–1.03]	5.00×10^{-13}	Howard DM
2	*SNRNP200, AC021188.1*	rs4435501	NR	0.13	[0.071–0.189] unit increase	4.00×10^{-6}	Hall LS
2	*AC018880.2 - AC013727.1*	rs2945451	0.239	0.01	[0.0046–0.01] unit decrease	1.00×10^{-7}	Howard DM
2	*FHL2 - AC018802.1*	rs111945524	0.045	0.02	[0.0096–0.0206] unit decrease	9.00×10^{-8}	Howard DM
2	*CNTNAP5*	rs11123030	0.491	1.03	[1.02–1.04]	1.00×10^{-9}	Cai N
2	*CNTNAP5*	rs11123030	0.510	0.01	[0.0036–0.0084] unit decrease	3.00×10^{-7}	Howard DM
2	*LINC01876*	rs1226412	0.792	1.02	[1.018–1.029]	3.00×10^{-18}	Howard DM
2	*LINC01876*	rs1226412	0.790	1.03	[1.02–1.04]	2.00×10^{-8}	Wray NR
2	*LY75-CD302, LY75*	rs2042772	0.255	0.03	[0.02–0.047] unit decrease	3.00×10^{-6}	Hyde CL
2	*RBMS1 - AC096656.1*	rs4455170	0.284	0.01	[0.0041–0.0091] unit increase	5.00×10^{-7}	Howard DM
2	*DNAJC10 - AC105396.1*	rs288342	NR	0.09	[0.051–0.129] unit decrease	7.00×10^{-6}	Hall LS
2	*CACYBPP2 - AC093639.3*	rs62195769	0.107	0.01	[0.0032–0.0072] unit increase	6.00×10^{-8}	Howard DM
2	*PCGEM1 - AC096647.1*	rs10174573	0.570	1.56	[1.39–1.74]	2.00×10^{-6}	GENDEP Investigators
2	*DNAH7 - STK17B*	rs139146062	0.024	1.07	[1.04–1.1]	9.00×10^{-6}	Coleman JRI
2	*DNAH7 - STK17B*	rs139146062	0.024	1.15	[1.09–1.2]	7.00×10^{-7}	Coleman JRI
2	*SF3B1*	rs699319	0.319	0.03	[0.02–0.044] unit increase	2.00×10^{-6}	Hyde CL
2	*MYOSLID, AC007879.1*	rs62188629	0.314	1.02	[1.012–1.022]	5.00×10^{-13}	Howard DM
2	*ERBB4, MTND2P23*	rs72948529	0.216	0.01	[0.0048–0.0102] unit increase	2.00×10^{-7}	Howard DM
2	*SP140L*	rs4973321	0.013	1.13	[1.08–1.19]	6.00×10^{-6}	Coleman JRI
2	*OR6B3 - OR9S24P*	rs116200762	0.067	1.13	[1.07–1.18]	9.00×10^{-6}	Coleman JRI
2	*MAB21L4*	rs78562453	NR			6.00×10^{-8}	Wong ML
3	*GRM7*	rs507385	NR	0.13	[0.071–0.189] unit decrease	9.00×10^{-6}	Hall LS
3	*GRM7*	rs9870680	0.440	1.19	[NR]	1.00×10^{-6}	Shyn SI
3	*ATG7*	rs6442260	NR	5.46	z score decrease	5.00×10^{-8}	Nagel M
3	*TBC1D5*	rs11708664	0.712	1.15	[1.09–1.21]	1.00×10^{-6}	Coleman JRI
3	*KCNH8*	rs116215823	0.011	1.07	[1.04–1.1]	7.00×10^{-6}	Coleman JRI
3	*RNU6-922P - RNU6-788P*	rs77685443	0.036	0.02	[0.011–0.025] unit increase	7.00×10^{-7}	Howard DM
3	*THRB*	rs138272922	0.011	1.16	[1.11–1.21]	5.00×10^{-8}	Coleman JRI
3	*RBMS3, AC098650.1*	rs1874256	NR	0.07	[0.031–0.109] unit increase	9.00×10^{-6}	Hall LS
3	*RBMS3, AC098650.1*	rs1874256	NR	0.10	[0.061–0.139] unit increase	4.00×10^{-6}	Hall LS
3	*CRTAP*	rs4478037	0.080	0.29	[0.19–0.39] unit increase	2.00×10^{-8}	Hall LS
3	*XYLB - ACVR2B-AS1*	rs73063271	NR	0.17	[0.092–0.248] unit decrease	6.00×10^{-6}	Hall LS
3	*ZKSCAN7-AS1 - SOCS5P3*	rs4346585	0.304	1.02	[1.01–1.02]	5.00×10^{-12}	Howard DM
3	*KLHDC8B - C3orf84*	rs13084037	0.226	1.02	[1.02–1.03]	2.00×10^{-17}	Howard DM
3	*ERC2*	rs7635184	0.602	0.01	[0.0039–0.0087] unit decrease	2.00×10^{-7}	Howard DM
3	*FHIT*	rs141954845	0.388	1.02	[1.014–1.022]	1.00×10^{-15}	Howard DM
3	*FHIT - PTPRG*	rs10514718	0.940	2.12		4.00×10^{-6}	Shi J
3	*PTPRG*	rs40493	NR	0.13	[0.071–0.189] unit increase	4.00×10^{-6}	Hall LS

Chr	Gene	SNP	Risk Allele Frequency	Beta	95% CI	P	First Author
3	*SYNPR*	rs73108923	NR	0.30	[0.16–0.44] unit decrease	6.00×10^{-6}	Hall LS
3	*ROBO2*	rs935526	NR	5.45	z score decrease	5.00×10^{-8}	Nagel M
3	*ROBO2*	rs775766	NR	5.66	z score increase	2.00×10^{-8}	Nagel M
3	*MRPS17P3 - ROBO1*	rs73117594	NR	5.66	z score increase	2.00×10^{-8}	Nagel M
3	*AC091212.1*	rs1481631	NR			2.00×10^{-6}	Liu Y
3	*RNU6-461P - RNU1-43P*	rs1304150	0.868	0.01	[0.0057–0.0131] unit increase	9.00×10^{-7}	Howard DM
3	*ZNF80 - TIGIT*	rs143445914	0.972	1.07	[1.04–1.1]	8.00×10^{-6}	Coleman JRI
3	*AC092691.3, AC092691.1*	rs6783233	0.283	1.02	[1.013–1.023]	1.00×10^{-13}	Howard DM
3	*AC092691.1*	rs66511648	0.284	1.03	[1.02–1.04]	2.00×10^{-8}	Cai N
3	*AC092691.1*	rs66511648	0.286	0.01	[0.0045–0.0095] unit increase	1.00×10^{-7}	Howard DM
3	*PARP15*	rs2173763	NR			6.00×10^{-6}	Sullivan PF
3	*RSRC1*	rs6441175	NR	6.04	z score decrease	2.00×10^{-9}	Nagel M
3	*RSRC1*	rs1095626	0.420	1.03	[1.02–1.03]	6.00×10^{-39}	Howard DM
3	*RSRC1*	rs1210358	0.615	1.03	[NR]	9.00×10^{-8}	Li X
3	*RSRC1*	rs7430565	0.420	1.03	[1.02–1.04]	3.00×10^{-9}	Wray NR
3	*AC106707.1*	rs1656369	0.670	0.03	[0.023–0.046] unit increase	1.00×10^{-8}	Hyde CL
3	*AC106707.1*	rs1656369	NR	5.84	z score decrease	5.00×10^{-9}	Nagel M
3	*IL12A-AS1*	rs6799788	0.248	1.63	[1.43–1.82]	1.00×10^{-6}	GENDEP Investigators
3	*AC069218.2 - AC069218.1*	rs13074924	0.360	1.18	[NR]	9.00×10^{-6}	Lewis CM
3	*LINC00578*	rs644695	0.880	1.35	[NR]	5.00×10^{-6}	Shyn SI
3	*LINC01014, AC117457.1, KCNMB2*	rs2291479	0.400	0.10	[0.061–0.139] unit decrease	1.00×10^{-6}	Hall LS
3	*SOX2-OT*	rs139388380	0.019	1.14	[1.08–1.19]	5.00×10^{-6}	Coleman JRI
3	*DVL3*	rs1969253	NR			5.00×10^{-6}	Sullivan PF
3	*C3orf70 - EHHADH-AS1*	rs7647854	0.160	1.19	[NR]	5.00×10^{-6}	Wray NR
3	*C3orf70 - EHHADH-AS1*	rs7647854	0.160	1.16	[1.11–1.21]	5.00×10^{-11}	Power RA
3	*AC026320.3*	rs4585146	0.650	1.16	[1.11–1.22]	6.00×10^{-6}	GENDEP Investigators
3	*AC026320.3*	rs4585146	0.362	0.22	[0.14–0.31] unit decrease	3.00×10^{-7}	GENDEP Investigators
3	*OPA1*	rs9838374	0.183	0.04	[0.022–0.053] unit increase	6.00×10^{-8}	Hyde CL
3	*LINC02038 - LINC02026*	rs604222	0.192	0.22	[0.12–0.31] unit increase	7.00×10^{-6}	GENDEP Investigators
4	*HTT*	rs7685686	0.575	1.02	[1.013–1.022]	6.00×10^{-15}	Howard DM
4	*LINC02473 - AC006390.1*	rs12500612	0.850	1.77	[1.51–2.02]	7.00×10^{-6}	GENDEP Investigators
4	*SLC30A9*	rs34215985	0.760	1.04	[1.03–1.05]	3.00×10^{-9}	Wray NR
4	*SLC30A9 - BEND4*	rs34937911	0.884	1.03	[1.018–1.032]	5.00×10^{-13}	Howard DM
4	*GABRB1*	rs13127214	NR	0.19	[0.11–0.27] unit increase	7.00×10^{-6}	Hall LS
4	*MTCO3P27 - MTCYBP16*	rs78825642	0.870	1.07	[1.04–1.1]	4.00×10^{-6}	Coleman JRI
4	*AC068721.1 - NPFFR2*	rs116569570	NR	0.16	[0.082–0.238] unit decrease	5.00×10^{-6}	Hall LS
4	*RASGEF1B*	rs1822818	0.870	1.77	[1.53–2.02]	6.00×10^{-6}	GENDEP Investigators
4	*AC073840.1*	rs9991694	0.767	1.16	[1.1–1.22]	5.00×10^{-7}	Coleman JRI
4	*UNC5C*	rs6822806	NR	0.18	[0.1–0.26] unit decrease	5.00×10^{-6}	Hall LS
4	*MRPS33P3 - RPF2P2*	rs116233998	NR	0.47	[0.27–0.67] unit decrease	7.00×10^{-6}	Hall LS
4	*KIAA1109*	rs77087420	NR	6.40	z score increase	2.00×10^{-10}	Nagel M
4	*KIAA1109*	rs45510091	0.947	1.05	[1.037–1.057]	8.00×10^{-21}	Howard DM
4	*AC133530.1 - AC097474.1*	rs2125000	0.307	1.43	[1.27–1.58]	3.00×10^{-6}	GENDEP Investigators
4	*TECRP2 - LINC02516*	rs6534441	0.333	0.18	[0.1–0.26] unit decrease	7.00×10^{-6}	GENDEP Investigators
4	*HSPA4L*	rs187279821	NR	0.42	[0.24–0.6] unit decrease	5.00×10^{-6}	Hall LS
4	*LINC02465 - LINC02479*	rs6818069	0.253	0.01	[0.0042–0.0096] unit increase	4.00×10^{-7}	Howard DM
4	*LINC02465 - LINC02479*	rs35553410	0.254	1.02	[1.01–1.02]	2.00×10^{-10}	Howard DM
4	*KRT18P54 - TARS2P1*	rs11099341	NR	0.09	[0.051–0.129] unit decrease	7.00×10^{-6}	Hall LS
4	*AC098859.2 - AC093766.1*	rs4475190	0.268	0.01	[0.0043–0.0093] unit decrease	5.00×10^{-7}	Howard DM
4	*PDGFC*	rs342318	NR	0.10	[0.061–0.139] unit increase	6.00×10^{-6}	Hall LS
4	*LINC02477 - RPS14P7*	rs115981586	NR	0.21	[0.11–0.31] unit increase	8.00×10^{-6}	Hall LS
4	*FAT1*	rs56390503	0.110	0.13	[0.071–0.189] unit increase	9.00×10^{-7}	Hall LS
4	*LINC02374 - AC097652.1*	rs4478239	NR			5.00×10^{-6}	Sullivan PF
5	*AC012629.1 - CTNND2*	rs6893200	NR	0.11	[0.071–0.149] unit decrease	5.00×10^{-6}	Hall LS
5	*AC012629.1 - CTNND2*	rs2964802	0.280	0.09	[0.051–0.129] unit increase	7.00×10^{-7}	Hall LS
5	*AC012629.1 - CTNND2*	rs2964802	NR	0.10	[0.061–0.139] unit increase	2.00×10^{-6}	Hall LS
5	*TRIO*	rs115054458	NR			3.00×10^{-7}	Wong ML
5	*MYO10*	rs17651119	0.990	3.18	[2.77–3.59]	2.00×10^{-8}	GENDEP Investigators
5	*HSPD1P15 - CDH18*	rs349475	0.290	0.18	[NR] unit increase	2.00×10^{-6}	Terracciano A
5	*CDH9*	rs1946473	NR	5.55	z score decrease	3.00×10^{-8}	Nagel M
5	*PURPL - AC093300.1*	rs12054895	0.275	0.24	[0.16–0.33] unit increase	3.00×10^{-8}	GENDEP Investigators
5	*LINC02119*	rs270545	0.690	1.37		1.00×10^{-6}	Shi J
5	*RN7SKP157 - KIF2A*	rs60157091	0.515	1.02	[1.016–1.025]	6.00×10^{-21}	Howard DM
5	*AC025445.1 - AC008558.1*	rs255428	NR	0.13	[0.071–0.189] unit increase	3.00×10^{-6}	Hall LS
5	*AC093270.3 - AC093270.1*	rs7713917	0.600	1.33	[1.19–1.49]	1.00×10^{-6}	Rietschel M
5	*VCAN - HAPLN1*	rs310501	0.330	1.16	[NR]	7.00×10^{-6}	Lewis CM
5	*LINC02144 - AC020930.1*	rs1673101	0.331	0.19	[0.11–0.27] unit decrease	4.00×10^{-6}	GENDEP Investigators
5	*AC114971.1 - TMEM161B*	rs1133152	0.132	1.05	[NR]	1.00×10^{-7}	Li X

Chr	Gene	SNP	Risk Allele Frequency	Beta	95% CI	P	First Author
5	*TMEM161B*	rs3099439	0.471	1.02	[1.02–1.03]	2.00×10^{-21}	Howard DM
5	*TMEM161B-AS1*	rs10514299	0.241	0.04	[0.031–0.059] unit increase	1.00×10^{-15}	Hyde CL
5	*LINC00461*	rs6882046	NR	5.66	z score decrease	1.00×10^{-8}	Nagel M
5	*MEF2C-AS2, AC008525.1*	rs2304607	NR	6.06	z score decrease	1.00×10^{-9}	Nagel M
5	*MEF2C-AS2*	rs454214	0.568	0.03	[0.022–0.045] unit decrease	1.00×10^{-9}	Hyde CL
5	*MEF2C-AS2*	rs454214	NR	5.49	z score decrease	4.00×10^{-8}	Nagel M
5	*FAM172A, POU5F2*	rs10061069	0.779	1.03	[1.02–1.03]	5.00×10^{-22}	Howard DM
5	*AC099520.1*	rs2028526	0.349	0.01	[0.0038–0.0086] unit increase	5.00×10^{-7}	Howard DM
5	*AC099520.1*	rs30266	0.328	1.04	[1.03–1.05]	4.00×10^{-12}	Cai N
5	*AC099520.1*	rs30266	0.334	1.08	[1.05–1.11]	2.00×10^{-7}	Coleman JRI
5	*AC099520.1*	rs30266	0.330	1.03	[1.028–1.037]	2.00×10^{-45}	Howard DM
5	*AC099520.1*	rs40465	0.332	0.01	[0.0053–0.0103] unit increase	4.00×10^{-10}	Howard DM
5	*AC099520.1*	rs161645	0.340			8.00×10^{-8}	Hek K
5	*AC099520.1*	rs323509	0.677	0.01	[0.0049–0.0099] unit decrease	3.00×10^{-9}	Howard DM
5	*FER*	rs10070411	0.778	1.08	[1.05–1.11]	7.00×10^{-7}	Coleman JRI
5	*FER*	rs17534846	0.656	1.14	[1.08–1.2]	8.00×10^{-6}	Coleman JRI
5	*LINC02200 - APC*	rs11743328	NR	0.10	[0.061–0.139] unit decrease	7.00×10^{-6}	Hall LS
5	*AC025465.1*	rs12513663	0.268	1.18	[1.11–1.26]	2.00×10^{-6}	GENDEP Investigators
5	*LINC02240*	rs116755193	0.620	1.03	[1.02–1.04]	7.00×10^{-9}	Wray NR
5	*AC112178.1 - AC109439.1*	rs35514087	0.048	1.09	[1.05–1.12]	2.00×10^{-7}	Coleman JRI
5	*CYSTM1, PFDN1*	rs13186198	0.536	1.07	[1.04–1.1]	9.00×10^{-6}	Coleman JRI
5	*PCDHA5*	rs3806843	0.449	0.03	[0.017–0.041] unit decrease	9.00×10^{-8}	Hyde CL
5	*PCDHA5*	rs3806843	NR	6.05	z score decrease	1.00×10^{-9}	Nagel M
5	*AC005753.1 - PCDH1*	rs166040	0.870	1.61	[1.41–1.8]	5.00×10^{-6}	GENDEP Investigators
5	*ARHGEF37 - AC022100.2*	rs17710780	0.860	1.66	[1.45–1.88]	4.00×10^{-6}	GENDEP Investigators
5	*AC008691.1*	rs10065906	0.332	1.17	[1.11–1.22]	2.00×10^{-6}	GENDEP Investigators
5	*AC008691.1*	rs10065906	0.308	1.47	[1.33–1.61]	5.00×10^{-8}	GENDEP Investigators
5	*AC008691.1*	rs10065906	0.362	1.53	[1.36–1.7]	2.00×10^{-6}	GENDEP Investigators
5	*AC109466.1*	rs7714851	0.520			8.00×10^{-16}	Amare AT
5	*AC109466.1*	rs4339373	0.510	1.03	[NR]	2.00×10^{-7}	Li X
5	*AC109466.1*	rs4543289	0.480	0.03	[0.02–0.044] unit decrease	1.00×10^{-8}	Hyde CL
5	*AC109466.1*	rs4543289	NR	5.50	z score decrease	4.00×10^{-8}	Nagel M
5	*AC109466.1*	rs4543289	NR	1.02	[1.01–1.03]	7.00×10^{-6}	Cai N
5	*AC109466.1*	rs10035449	0.536	0.01	[0.0037–0.0085] unit increase	2.00×10^{-7}	Howard DM
5	*AC109466.1*	rs11135349	0.529	1.03	[1.02–1.03]	2.00×10^{-30}	Howard DM
5	*AC109466.1*	rs11135349	0.520	1.03	[1.02–1.04]	1.00×10^{-9}	Wray NR
5	*AC109466.1*	rs10476497	0.534	1.07	[1.04–1.1]	5.00×10^{-6}	Coleman JRI
5	*LINC01938*	rs564483817	0.376	0.01	[0.0038–0.0086] unit decrease	3.00×10^{-7}	Howard DM
5	*TENM2*	rs2098651	NR	6.34	z score increase	2.00×10^{-10}	Nagel M
5	*TENM2*	rs4869056	0.370	1.03	[1.02–1.04]	7.00×10^{-9}	Wray NR
5	*TENM2*	rs62383381	0.164	0.01	[0.0047–0.0109] unit increase	9.00×10^{-7}	Howard DM
5	*PANK3*	rs35693458	0.148	1.14	[1.08–1.19]	8.00×10^{-6}	Coleman JRI
5	*MGAT4B, MGAT4B*	rs272440	0.999	0.48	[0.27–0.69] unit decrease	6.00×10^{-7}	Hyde CL
6	*LY86-AS1, LY86*	rs2326810	0.920	1.41	[NR]	7.00×10^{-6}	Shyn SI
6	*OFCC1*	rs12210761	0.068	2.45	[2.06–2.83]	6.00×10^{-6}	GENDEP Investigators
6	*AL024498.2, TMEM14B, MAK*	rs114927772	NR	0.35	[0.21–0.49] unit increase	3.00×10^{-6}	Hall LS
6	*GMPR*	rs10484358	0.083	0.43	[0.25–0.6] unit decrease	1.00×10^{-6}	GENDEP Investigators
6	*AL133270.1 - AL139093.1*	rs9466930	0.215	1.50	[1.32–1.68]	4.00×10^{-6}	GENDEP Investigators
6	*DCDC2*	rs12189861	0.105	0.01	[0.0058–0.0132] unit increase	7.00×10^{-7}	Howard DM
6	*DCDC2*	rs12205083	0.104	1.05	[1.04–1.07]	6.00×10^{-10}	Cai N
6	*SCGN - H2AC1*	rs68006638	0.094	0.01	[0.0063–0.0141] unit decrease	6.00×10^{-7}	Howard DM
6	*H2BC4*	rs144735131	NR	0.40	[0.24–0.56] unit decrease	2.00×10^{-6}	Hall LS
6	*BTN3A2*	rs13218591	NR	5.61	z score increase	2.00×10^{-8}	Nagel M
6	*BTN3A1*	rs75782365	0.890	1.05	[1.04–1.07]	1.00×10^{-9}	Cai N
6	*BTN3A1*	rs75782365	0.105	0.01	[0.0075–0.0149] unit decrease	6.00×10^{-9}	Howard DM
6	*BTN2A1*	rs3799380	NR	5.77	z score increase	8.00×10^{-9}	Nagel M
6	*ABT1*	rs45527431	0.105	0.01	[0.0078–0.0152] unit decrease	2.00×10^{-9}	Howard DM
6	*AL513548.1 - GUSBP2*	rs35144506	0.118	0.01	[0.0077–0.0151] unit decrease	5.00×10^{-9}	Howard DM
6	*LINC00240*	rs66975207	0.889	1.08	NR	1.00×10^{-9}	Cai N
6	*LINC00240*	rs66975207	0.106	0.01	[0.0074–0.0148] unit decrease	7.00×10^{-9}	Howard DM
6	*VN1R11P - RNU2-62P*	rs55834529	0.106	0.01	[0.0077–0.0151] unit decrease	3.00×10^{-9}	Howard DM
6	*MCFD2P1 - ZNF184*	rs2064219	NR	6.15	z score increase	8.00×10^{-10}	Nagel M
6	*MCFD2P1 - ZNF184*	rs7772160	0.522	1.04	[1.03–1.05]	3.00×10^{-12}	Cai N
6	*AL021918.5*	rs7749305	0.121	0.01	[0.0084–0.0154] unit decrease	4.00×10^{-11}	Howard DM
6	*RNU6-471P - AL031229.1*	rs35715914	0.108	0.01	[0.0088–0.0162] unit decrease	8.00×10^{-11}	Howard DM
6	*H2BC15*	rs200990	NR	6.26	z score increase	4.00×10^{-10}	Nagel M
6	*H1-5 - H3C11*	rs200949	0.874	1.04	[1.037–1.051]	2.00×10^{-37}	Howard DM
6	*H4C13 - H3C12*	rs483143	0.128	0.01	[0.0086–0.0156] unit decrease	8.00×10^{-12}	Howard DM

Chr	Gene	SNP	Risk Allele Frequency	Beta	95% CI	P	First Author
6	*ZSCAN16-AS1, ZSCAN12P1*	rs13197574	0.110	0.01	[0.0091–0.0165] unit decrease	1.00×10^{-11}	Howard DM
6	*AL358933.1*	rs4713145	NR	1.03	[1.02–1.04]	2.00×10^{-7}	Cai N
6	*AL358933.1*	rs4713145	0.756	1.06	NR	5.00×10^{-11}	Cai N
6	*ZKSCAN3 - ZSCAN12*	rs35017208	0.111	0.01	[0.0096–0.017] unit decrease	1.00×10^{-12}	Howard DM
6	*GPX6, GPX6*	rs13191038	0.110	0.01	[0.0094–0.0168] unit decrease	4.00×10^{-12}	Howard DM
6	*AL121932.2 - RPSAP2*	rs13194504	0.112	0.01	[0.009–0.0164] unit decrease	1.00×10^{-11}	Howard DM
6	*AL121932.2 - RPSAP2*	rs7775835	NR	5.59	z score decrease	2.00×10^{-8}	Nagel M
6	*NOP56P1 - AL662890.1*	rs3135296	NR	1.10	[1.06–1.15]	5.00×10^{-7}	Cai N
6	*NOP56P1 - AL662890.1*	rs3135296	0.880	1.06	[1.04–1.07]	4.00×10^{-12}	Cai N
6	*NOP56P1 - AL662890.1*	rs3135296	NR	1.14	[1.08–1.19]	1.00×10^{-6}	Cai N
6	*NOP56P1 - AL662890.1*	rs9257248	0.114	0.01	[0.0086–0.016] unit decrease	4.00×10^{-11}	Howard DM
6	*HCG15*	rs3129791	0.115	0.01	[0.0088–0.0162] unit decrease	2.00×10^{-11}	Howard DM
6	*AL645937.1 - AL645937.4*	rs3129120	NR	1.10	[1.06–1.15]	4.00×10^{-7}	Cai N
6	*AL645937.1 - AL645937.4*	rs3129120	0.876	1.08	NR	1.00×10^{-10}	Cai N
6	*AL645937.4*	rs4947263	0.118	0.01	[0.0092–0.0162] unit decrease	3.00×10^{-12}	Howard DM
6	*AL672167.1*	rs9257681	0.115	0.01	[0.0094–0.0168] unit decrease	2.00×10^{-12}	Howard DM
6	*OR11A1*	rs2523443	0.119	0.01	[0.0095–0.0165] unit decrease	1.00×10^{-12}	Howard DM
6	*GABBR1*	rs926552	0.860	1.11	[1.07–1.14]	4.00×10^{-8}	Cai N
6	*GABBR1*	rs1233393	0.122	0.01	[0.0094–0.0164] unit decrease	8.00×10^{-13}	Howard DM
6	*AL645939.4 - HLA-V*	rs9258375	0.122	0.01	[0.0094–0.0168] unit decrease	1.00×10^{-12}	Howard DM
6	*HCG9*	rs3132685	0.130	0.01	[0.0096–0.0166] unit decrease	2.00×10^{-13}	Howard DM
6	*ZNRD1ASP*	rs3094146	0.870	1.15	[1.10–1.20]	5.00×10^{-8}	Cai N
6	*ZNRD1ASP*	rs3115631	NR	1.10	[1.06–1.15]	4.00×10^{-7}	Cai N
6	*ZNRD1ASP*	rs3115631	0.874	1.06	[1.04–1.08]	3.00×10^{-13}	Cai N
6	*TRIM31*	rs2517601	0.133	0.01	[0.0093–0.0159] unit decrease	5.00×10^{-13}	Howard DM
6	*PAIP1P1*	rs2517622	0.863	1.08	NR	4.00×10^{-11}	Cai N
6	*PAIP1P1*	rs2517622	NR	1.12	[1.07–1.18]	3.00×10^{-6}	Cai N
6	*UBQLN1P1 - MICC*	rs3130116	0.139	0.01	[0.0091–0.0157] unit decrease	6.00×10^{-13}	Howard DM
6	*HLA-E - LINC02569*	rs3131115	0.672	1.08	[1.05–1.12]	1.00×10^{-7}	Coleman JRI
6	*HLA-E - LINC02569*	rs28894081	0.066	0.01	[0.0079–0.0173] unit increase	1.00×10^{-7}	Howard DM
6	*GNL1*	rs2524222	0.220	0.03	[0.02–0.047] unit increase	1.00×10^{-6}	Hyde CL
6	*HCG20*	rs2284174	0.219	0.01	[0.0075–0.0129] unit decrease	1.00×10^{-12}	Howard DM
6	*HCG20*	rs115507122	0.820	1.04	[1.03–1.05]	3.00×10^{-11}	Wray NR
6	*NAPGP2 - MUC21*	rs3131781	0.145	0.01	[0.0064–0.013] unit decrease	9.00×10^{-9}	Howard DM
6	*NAPGP2 - MUC21*	rs9368649	0.140			1.00×10^{-8}	Amare AT
6	*RNU6-1133P - C6orf15*	rs28732092	0.079	0.01	[0.0067–0.0153] unit increase	5.00×10^{-7}	Howard DM
6	*HLA-B*	rs1625792	0.853	1.04	[1.03–1.05]	5.00×10^{-8}	Cai N
6	*HLA-B*	rs2596505	0.168	0.01	[0.0059–0.0121] unit decrease	1.00×10^{-8}	Howard DM
6	*PRRC2A*	rs2736155	0.478	0.01	[0.0035–0.0083] unit increase	6.00×10^{-7}	Howard DM
6	*CFB*	rs537160	0.639	0.01	[0.0039–0.0087] unit increase	3.00×10^{-7}	Howard DM
6	*HLA-DRB1 - HLA-DQA1*	rs535777	NR	1.04	[1.02–1.05]	5.00×10^{-6}	Cai N
6	*HLA-DRB1 - HLA-DQA1*	rs535777	0.862	1.08	NR	1.00×10^{-9}	Cai N
6	*MLN - LINC01016*	rs56366527	0.386	0.01	[0.0038–0.0086] unit decrease	2.00×10^{-7}	Howard DM
6	*MLN - LINC01016*	rs6922076	0.546	1.07	[1.04–1.1]	7.00×10^{-6}	Coleman JRI
6	*PPIL1*	rs236346	0.905	1.05	[1.03–1.07]	1.00×10^{-8}	Cai N
6	*GLO1*	rs116078874	NR	0.47	[0.27–0.67] unit increase	4.00×10^{-6}	Hall LS
6	*TUBBP9 - AL139275.2*	rs10947863	0.747	1.08	[1.05–1.11]	2.00×10^{-6}	Coleman JRI
6	*AL109615.4 - MRPL14*	rs7742824	0.224	1.19	[1.11–1.26]	9.00×10^{-6}	GENDEP Investigators
6	*FTH1P5 - AL158050.2*	rs1575671	0.139	1.07	[1.04–1.1]	7.00×10^{-6}	Coleman JRI
6	*FTH1P5 - AL158050.2*	rs116100277	0.014	1.07	[1.04–1.1]	1.00×10^{-6}	Coleman JRI
6	*ADH5P4 - NUFIP1P1*	rs9363467	0.604	1.02	[1.011–1.021]	3.00×10^{-12}	Howard DM
6	*ADH5P4 - NUFIP1P1*	rs76736233	0.134	0.01	[0.0055–0.0125] unit increase	3.00×10^{-7}	Howard DM
6	*ADH5P4 - NUFIP1P1*	rs9345737	0.561	1.03	[1.02–1.04]	3.00×10^{-9}	Cai N
6	*AL713998.3, AL713998.4*	rs140992857	0.031	1.07	[1.04–1.1]	8.00×10^{-6}	Coleman JRI
6	*KCNQ5*	rs112348907	0.296	0.01	[0.0048–0.0098] unit increase	2.00×10^{-8}	Howard DM
6	*CASP8AP2*	rs111404313	NR	0.32	[0.18–0.46] unit decrease	4.00×10^{-6}	Hall LS
6	*AL591519.1*	rs147643734	0.960	1.07	[1.04–1.1]	6.00×10^{-6}	Coleman JRI
6	*AL607077.1 - AL034348.1*	rs71560357	0.010	1.07	[1.04–1.1]	8.00×10^{-6}	Coleman JRI
6	*UFL1-AS1*	rs116842147	0.011	1.08	[1.05–1.1]	6.00×10^{-7}	Coleman JRI
6	*AL078603.1 - MIR548AI*	rs10457592	0.761	0.04	[0.023–0.051] unit decrease	6.00×10^{-6}	Hyde CL
6	*AL078603.1 - MIR548AI*	rs9402472	0.240	1.03	[1.02–1.04]	3.00×10^{-8}	Wray NR
6	*AL080285.1 - SIM1*	rs17792983	0.305	0.03	[0.016–0.044] unit decrease	6.00×10^{-8}	Hyde CL
6	*AL133338.2 - GRIK2*	rs7758630	0.595	1.02	[1.01–1.02]	8.00×10^{-14}	Howard DM
6	*GRIK2*	rs112904378	0.035	1.07	[1.04–1.1]	9.00×10^{-6}	Coleman JRI
6	*LIN28B-AS1*	rs1933802	0.546	1.02	[1.02–1.02]	1.00×10^{-19}	Howard DM
6	*LIN28B*	rs1475120	0.452	0.03	[0.017–0.041] unit decrease	4.00×10^{-9}	Hyde CL
6	*DDO*	rs10447426	0.043	0.02	[0.0094–0.0212] unit decrease	3.00×10^{-7}	Howard DM
6	*AL355337.1*	rs2876520	0.467	0.01	[0.0038–0.0086] unit increase	2.00×10^{-7}	Howard DM

Chr	Gene	SNP	Risk Allele Frequency	Beta	95% CI	P	First Author
6	*AL355337.1*	rs2876520	0.473	1.02	[1.01–1.02]	3.00×10^{-17}	Howard DM
6	*SAMD5*	rs725616	0.364	1.01	[1.009–1.018]	1.00×10^{-9}	Howard DM
6	*CCDC170*	rs149389640	NR	6.18	z score increase	7.00×10^{-10}	Nagel M
6	*SLC22A2*	rs117901488	NR	0.20	[0.12–0.28] unit increase	6.00×10^{-6}	Hall LS
6	*AL358972.1 - AL450345.2*	rs4709845	0.850	1.21	[NR]	9.00×10^{-6}	Lewis CM
6	*AL358972.1 - AL450345.2*	rs2029865	0.547	1.02	[1.01–1.02]	4.00×10^{-17}	Howard DM
6	*AL358972.1 - AL450345.2*	rs10806805	0.540	0.03	[0.015–0.038] unit increase	6.00×10^{-6}	Hyde CL
7	*MAD1L1, AC069288.1*	rs2056477	NR	5.47	z score decrease	4.00×10^{-8}	Nagel M
7	*AC069288.1, MAD1L1*	rs56072378	0.220	0.00	[0.0021–0.0049] unit decrease	9.00×10^{-7}	Howard DM
7	*MAD1L1, AC069288.1*	rs3823624	0.807	1.03	[1.024–1.036]	2.00×10^{-26}	Howard DM
7	*THSD7A - TMEM106B*	rs3807866	0.410	1.04	[1.03–1.05]	5.00×10^{-13}	Cai N
7	*THSD7A - TMEM106B*	rs3807866	0.398	1.08	[1.05–1.11]	1.00×10^{-7}	Coleman JRI
7	*THSD7A - TMEM106B*	rs3807865	0.412	0.01	[0.0058–0.0106] unit increase	7.00×10^{-12}	Howard DM
7	*TMEM106B*	rs2043539	0.418	1.02	[1.018–1.027]	8.00×10^{-24}	Howard DM
7	*TMEM106B*	rs3823612	NR	6.58	z score increase	5.00×10^{-11}	Nagel M
7	*TMEM106B*	rs10950398	0.410	1.03	[1.02–1.04]	3.00×10^{-8}	Wray NR
7	*TMEM106B*	rs5011432	0.412	0.01	[0.0048–0.0098] unit increase	2.00×10^{-8}	Howard DM
7	*AC011287.1 - AC005019.2*	rs9648182	NR	0.11	[0.051–0.169] unit decrease	8.00×10^{-6}	Hall LS
7	*AC011287.1 - AC005019.2*	rs9648182	0.120	0.17	[0.11–0.23] unit decrease	9.00×10^{-8}	Hall LS
7	*DGKB*	rs10227749	NR	0.12	[0.061–0.179] unit increase	4.00×10^{-6}	Hall LS
7	*AC007091.1*	rs12667733	0.496	0.00	[0.002–0.0044] unit decrease	6.00×10^{-8}	Howard DM
7	*SP4*	rs17144465	0.030	1.56	[NR]	8.00×10^{-7}	Shyn SI
7	*SP4*	rs17144465	0.040	1.82		6.00×10^{-6}	Shi J
7	*CDCA7L - RAPGEF5*	rs10247376	0.028	1.07	[1.04–1.1]	8.00×10^{-6}	Coleman JRI
7	*GSDME*	rs2721811	NR	5.50	z score decrease	4.00×10^{-8}	Nagel M
7	*ADCYAP1R1 - AC006466.1*	rs1558477				3.00×10^{-7}	Aragam N
7	*VPS41*	rs10274968	NR	5.71	z score increase	1.00×10^{-8}	Nagel M
7	*AC073115.2*	rs2462686	0.351	1.49	[1.31–1.66]	9.00×10^{-6}	GENDEP Investigators
7	*AC073873.1 - GALNT17*	rs1525293	0.660	1.70	[1.48–1.92]	2.00×10^{-6}	GENDEP Investigators
7	*MAGI2*	rs535355	0.378	1.15	[1.1–1.21]	7.00×10^{-7}	Coleman JRI
7	*CD36 - SEMA3C*	rs111747875	0.019	1.07	[1.04–1.1]	3.00×10^{-6}	Coleman JRI
7	*CACNA2D1*	rs17176546	0.040	0.26	[0.16–0.36] unit increase	8.00×10^{-7}	Hall LS
7	*PCLO*	rs2715148				1.00×10^{-6}	Aragam N
7	*PCLO*	rs2247523	0.468	1.02	[1.01–1.02]	9.00×10^{-14}	Howard DM
7	*PCLO*	rs2247523	NR			5.00×10^{-6}	Liu Y
7	*PCLO*	rs2715157	0.520	1.27	[1.16–1.37]	3.00×10^{-8}	Mbarek H
7	*PCLO - AC079799.1*	rs16887442	0.435	1.02	[1.011–1.019]	1.00×10^{-11}	Howard DM
7	*AC002386.1 - AC073071.1*	rs58104186	0.469	1.02	[1.015–1.024]	4.00×10^{-18}	Howard DM
7	*AC002386.1 - AC073071.1*	rs12666117	0.470	1.03	[1.02–1.04]	1.00×10^{-8}	Wray NR
7	*LINC01392*	rs6466512	0.417	0.01	[0.0035–0.0083] unit decrease	8.00×10^{-7}	Howard DM
7	*CTTNBP2*	rs7807677	0.551	1.02	[1.017–1.026]	5.00×10^{-22}	Howard DM
7	*AC007568.1 - AC003084.1*	rs2402273	0.409	0.01	[0.0048–0.0096] unit increase	2.00×10^{-9}	Howard DM
7	*AC006148.1*	rs138879850	0.991	1.08	[1.05–1.11]	2.00×10^{-7}	Coleman JRI
7	*GRM8*	rs17864092	0.900	0.17	[NR] unit decrease	6.00×10^{-6}	Terracciano A
7	*TSPAN33*	rs34534607	0.201	1.07	[1.04–1.1]	6.00×10^{-6}	Coleman JRI
7	*MKLN1*	rs10265216	0.280	1.19	[NR]	3.00×10^{-6}	Shyn SI
7	*OR6W1P - PIP*	rs28672333	0.073	1.16	[1.1–1.22]	2.00×10^{-7}	Coleman JRI
7	*RNY4 - RNY3*	rs1676905	0.926	1.07	[1.04–1.1]	3.00×10^{-6}	Coleman JRI
7	*KRBA1*	rs748441912	NR			5.00×10^{-7}	Wong ML
7	*AC006348.1 - AC079809.1*	rs144369419	0.011	0.03	[0.018–0.042] unit decrease	5.00×10^{-7}	Howard DM
8	*ATP6V1B2*	rs1106634	0.140	1.30	[NR]	7.00×10^{-7}	Shyn SI
8	*SLC25A37 - RNU4-71P*	rs950893	0.280	4.76	z score decrease	2.00×10^{-6}	Peterson RE
8	*SLC25A37 - RNU4-71P*	rs950893	0.280	5.79	z score decrease	7.00×10^{-9}	Peterson RE
8	*CDCA2 - AC009623.1*	rs2012485	0.033	0.08	[0.046–0.108] unit increase	3.00×10^{-6}	Hyde CL
8	*NRG1*	rs117715236	NR	0.24	[0.14–0.34] unit decrease	4.00×10^{-6}	Hall LS
8	*AC090993.1 - LINC01288*	rs139340185	0.010	1.07	[1.04–1.1]	7.00×10^{-6}	Coleman JRI
8	*PCMTD1*	rs9298467	0.809	0.04	[0.023–0.054] unit decrease	2.00×10^{-7}	Hyde CL
8	*CHD7*	rs4289855	0.228	1.04	[NR]	2.00×10^{-7}	Li X
8	*CYP7B1*	rs7837935	0.848	1.02	[1.02–1.03]	1.00×10^{-13}	Howard DM
8	*TRIM55*	rs7835340	NR	0.09	[0.051–0.129] unit increase	3.00×10^{-6}	Hall LS
8	*AC022730.2 - AC022730.3*	rs67436663	0.760	1.01	[1.01–1.02]	3.00×10^{-8}	Howard DM
8	*AC103409.1*	rs12547341	NR	0.08	[0.041–0.119] unit increase	5.00×10^{-6}	Hall LS
8	*C8orf37-AS1*	rs145478122	NR	0.30	[0.16–0.44] unit decrease	9.00×10^{-6}	Hall LS
8	*RNF19A - AP001574.1*	rs79802045	NR	0.25	[0.13–0.37] unit decrease	5.00×10^{-6}	Hall LS
8	*ZFPM2*	rs4565433	0.159	1.07	[1.04–1.1]	2.00×10^{-6}	Coleman JRI
8	*TRPS1*	rs2721937	NR			8.00×10^{-6}	Sullivan PF
8	*KCNQ3*	rs186555608	0.985	1.08	[1.04–1.11]	1.00×10^{-6}	Coleman JRI
8	*AC105213.1*	rs6995581	0.209	1.07	[1.04–1.11]	3.00×10^{-6}	Coleman JRI

Chr	Gene	SNP	Risk Allele Frequency	Beta	95% CI	P	First Author
8	*TRAPPC9*	rs79225303	NR	0.17	[0.092–0.248] unit decrease	2.00×10^{-6}	Hall LS
9	*DMRT1*	rs364477	0.215	1.69	[1.47–1.92]	4.00×10^{-6}	GENDEP Investigators
9	*CARM1P1*	rs7044150	0.385	0.03	[0.018–0.042] unit decrease	4.00×10^{-8}	Hyde CL
9	*CARM1P1*	rs1354115	0.624	1.02	[1.014–1.023]	4.00×10^{-17}	Howard DM
9	*CARM1P1*	rs1354115	0.620	1.03	[1.02–1.04]	2.00×10^{-8}	Wray NR
9	*AKAP8P1 - AL451129.1*	rs10809385	0.404	0.01	[0.0041–0.0089] unit decrease	6.00×10^{-8}	Howard DM
9	*AKAP8P1 - AL451129.1*	rs10959631	NR	0.10	[0.061–0.139] unit decrease	4.00×10^{-6}	Hall LS
9	*AKAP8P1 - AL451129.1*	rs10959631	0.200	0.12	[0.081–0.159] unit decrease	8.00×10^{-7}	Hall LS
9	*AKAP8P1 - AL451129.1*	rs11515172	0.784	1.09	[1.06–1.12]	4.00×10^{-8}	Coleman JRI
9	*AL355672.1 - AL592227.1*	rs56116032	0.222	0.01	[0.0049–0.0103] unit decrease	8.00×10^{-8}	Howard DM
9	*AL355672.1 - AL592227.1*	rs1982277	0.759	1.02	[1.016–1.026]	1.00×10^{-15}	Howard DM
9	*AL355672.1 - AL592227.1*	rs10959913	0.760	1.03	[1.02–1.04]	5.00×10^{-9}	Wray NR
9	*AL355672.1 - AL592227.1*	rs10959926	0.218	0.01	[0.0051–0.0105] unit decrease	6.00×10^{-8}	Howard DM
9	*AL353595.1*	rs10960103	0.227	0.01	[0.0049–0.0103] unit decrease	5.00×10^{-8}	Howard DM
9	*AL162725.2*	rs263645	0.544	1.02	[1.013–1.022]	7.00×10^{-16}	Howard DM
9	*AL162725.2*	rs263575	0.460	0.01	[0.0042–0.009] unit decrease	2.00×10^{-8}	Howard DM
9	*AL162725.2*	rs393488	0.531	1.03	[1.02–1.04]	2.00×10^{-10}	Cai N
9	*ELAVL2*	rs3793577	NR	6.99	z score decrease	3.00×10^{-12}	Nagel M
9	*ELAVL2*	rs3793577	0.534	1.02	[1.02–1.03]	6.00×10^{-22}	Howard DM
9	*ELAVL2*	rs10811965	NR	5.63	z score decrease	2.00×10^{-8}	Nagel M
9	*RN7SKP120 - AL353730.1*	rs59283172	0.893	1.02	[1.01–1.02]	1.00×10^{-6}	Howard DM
9	*RN7SKP120 - AL353730.1*	rs12057031	0.892	1.05	[1.03–1.07]	5.00×10^{-9}	Cai N
9	*RN7SKP120 - AL353730.1*	rs73474148	0.110	0.01	[0.0065–0.0139] unit decrease	7.00×10^{-8}	Howard DM
9	*HSPA8P17 - AL354854.1*	rs34653192	0.680	1.02	[1.02–1.02]	3.00×10^{-16}	Howard DM
9	*B4GALT1-AS1 - SPINK4*	rs62544433	0.114	0.01	[0.0064–0.0142] unit increase	4.00×10^{-7}	Howard DM
9	*AL356218.2 - AQP3*	rs10758202	NR	0.14	[0.081–0.199] unit increase	2.00×10^{-6}	Hall LS
9	*PAX5*	rs7030813	0.374	0.01	[0.0038–0.0086] unit increase	4.00×10^{-7}	Howard DM
9	*PAX5*	rs7030813	0.374	1.03	[1.021–1.03]	9.00×10^{-30}	Howard DM
9	*AL161781.2, PAX5*	rs6476606	0.637	0.03	[0.016–0.039] unit decrease	1.00×10^{-8}	Hyde CL
9	*TRPM3*	rs188386326	0.014	1.07	[1.04–1.1]	8.00×10^{-6}	Coleman JRI
9	*FAM120A*	rs12376738	NR	5.65	z score increase	2.00×10^{-8}	Nagel M
9	*ASTN2*	rs10817969	0.717	1.02	[1.012–1.022]	1.00×10^{-12}	Howard DM
9	*ASTN2*	rs7856424	0.710	1.03	[1.02–1.04]	9.00×10^{-9}	Wray NR
9	*AL160272.2, TLR4*	rs913930	0.357	1.02	[1.02–1.03]	1.00×10^{-21}	Howard DM
9	*AL160272.2*	rs2149351	NR	5.55	z score increase	3.00×10^{-8}	Nagel M
9	*AC006288.1 - LINC01613*	rs2781093	0.723	1.07	[1.04–1.1]	9.00×10^{-6}	Coleman JRI
9	*DENND1A*	rs2670139	0.239	1.02	[1.01–1.02]	2.00×10^{-12}	Howard DM
9	*DENND1A*	rs7029033	0.070	1.05	[1.03–1.07]	3.00×10^{-8}	Wray NR
9	*DENND1A*	rs12115299	0.069	0.05	[0.029–0.076] unit increase	2.00×10^{-6}	Hyde CL
9	*SLC2A8*	rs115668237	NR			6.00×10^{-6}	Wong ML
9	*AK8*	rs12552369	0.494	1.16	[1.1–1.21]	8.00×10^{-6}	GENDEP Investigators
10	*ADARB2 - AL355597.1*	rs997934	0.380	1.02	[1.012–1.021]	5.00×10^{-13}	Howard DM
10	*AL713851.1*	rs78245978	NR	0.43	[0.25–0.61] unit increase	6.00×10^{-6}	Hall LS
10	*RSU1*	rs61842316	NR	0.27	[0.15–0.39] unit increase	3.00×10^{-6}	Hall LS
10	*LINC01517 - LYZL1*	rs1612122	0.480	1.17	[NR]	7.00×10^{-6}	Shyn SI
10	*ITGB1-DT*	rs11009175	0.170	0.16	[NR] unit increase	5.00×10^{-6}	Terracciano A
10	*LINC02629 - PARD3*	rs1780436				3.00×10^{-6}	Aragam N
10	*LINC02629 - PARD3*	rs1780436	NR			7.00×10^{-6}	Liu Y
10	*RASGEF1A*	rs2460546	NR	0.29	[0.17–0.41] unit increase	4.00×10^{-6}	Hall LS
10	*AC069545.1 - ZWINT*	rs117778141	NR	0.50	[0.3–0.7] unit decrease	1.00×10^{-6}	Hall LS
10	*CTNNA3*	rs10762080	0.586	0.01	[0.0036–0.0084] unit decrease	6.00×10^{-7}	Howard DM
10	*RNU6-523P - RPL12P8*	rs12415800	0.460	5.02	z score increase	5.00×10^{-7}	Peterson RE
10	*RNU6-523P - RPL12P8*	rs12415800	0.460	4.66	z score increase	3.00×10^{-6}	Peterson RE
10	*DDX50 - DDX21*	rs2017305	0.920	0.22	[NR] unit decrease	9.00×10^{-6}	Terracciano A
10	*AC012560.2*	rs164241	0.089	1.14	[1.09–1.2]	2.00×10^{-6}	Coleman JRI
10	*GRID1*	rs55940619	0.923	1.15	[1.09–1.21]	4.00×10^{-6}	Coleman JRI
10	*SORCS3*	rs11599236	0.592	1.03	[1.02–1.04]	3.00×10^{-8}	Cai N
10	*SORCS3*	rs11599236	0.409	0.01	[0.0041–0.0089] unit decrease	8.00×10^{-8}	Howard DM
10	*SORCS3*	rs61867293	0.800	1.04	[1.03–1.05]	7.00×10^{-10}	Wray NR
10	*SORCS3*	rs1021363	0.642	0.01	[0.0046–0.0094] unit decrease	1.00×10^{-8}	Howard DM
10	*SORCS3*	rs1021363	0.355	1.02	[1.018–1.027]	4.00×10^{-23}	Howard DM
10	*SORCS3*	rs10786831	0.601	0.03	[0.018–0.041] unit decrease	8.00×10^{-9}	Hyde CL
10	*SORCS3*	rs10786831	NR	1.03	[1.015–1.036]	2.00×10^{-6}	Cai N
10	*SORCS3*	rs1961639	NR	5.89	z score decrease	4.00×10^{-9}	Nagel M
10	*ENO4*	rs12240686	NR	0.18	[0.1–0.26] unit decrease	6.00×10^{-6}	Hall LS
10	*MCMBP*	rs113416614	0.068	1.07	[1.04–1.1]	8.00×10^{-6}	Coleman JRI
10	*LHPP*	rs35936514	0.260	4.78	z score decrease	2.00×10^{-6}	Peterson RE
11	*MUC5B*	rs56293203	NR			2.00×10^{-9}	Wong ML

Chr	Gene	SNP	Risk Allele Frequency	Beta	95% CI	P	First Author
11	*EIF3F*	rs12420464	0.040	0.38	[NR] unit decrease	3.00×10^{-6}	Terracciano A
11	*METTL15 - LINC02758*	rs10767735	0.395	0.01	[0.0036–0.0084] unit decrease	7.00×10^{-7}	Howard DM
11	*DCDC1, AL122014.1*	rs620339	0.593	0.03	[0.018–0.042] unit decrease	8.00×10^{-8}	Hyde CL
11	*DCDC1*	rs1448938	0.417	1.02	[1.013–1.022]	4.00×10^{-15}	Howard DM
11	*AL035078.4, PAUPAR*	rs1806153	0.220	1.04	[1.03–1.05]	1.00×10^{-9}	Wray NR
11	*AL035078.4, PAUPAR*	rs1806152	0.172	0.04	[0.026–0.057] unit increase	4.00×10^{-7}	Hyde CL
11	*CCDC73*	rs143864773	0.931	1.08	[1.05–1.11]	1.00×10^{-6}	Coleman JRI
11	*AC090692.2 - KRT18P14*	rs11033303	0.370	0.09	[0.051–0.129] unit increase	2.00×10^{-7}	Hall LS
11	*AC090692.2 - KRT18P14*	rs11033303	0.370	0.11	[0.071–0.149] unit increase	6.00×10^{-7}	Hall LS
11	*ZDHHC5*	rs10896636	NR	5.96	z score decrease	3.00×10^{-9}	Nagel M
11	*OR5BA1P - OR5AZ1P*	rs2509805	0.321	1.02	[1.015–1.024]	5.00×10^{-16}	Howard DM
11	*MS4A1 - MS4A12*	rs17615103	NR	0.22	[0.12–0.32] unit increase	6.00×10^{-6}	Hall LS
11	*MS4A13*	rs150439352	NR	0.47	[0.27–0.67] unit increase	4.00×10^{-6}	Hall LS
11	*DAGLA*	rs198457	0.808	1.02	[1.02–1.03]	5.00×10^{-14}	Howard DM
11	*LTBP3*	rs58621819	0.210	1.02	[1.01–1.02]	2.00×10^{-10}	Howard DM
11	*CCS*	rs1791684	0.526	0.01	[0.0037–0.0085] unit increase	3.00×10^{-7}	Howard DM
11	*LTO1 - FGF19*	rs80208956	NR	0.30	[0.16–0.44] unit increase	6.00×10^{-6}	Hall LS
11	*SHANK2*	rs7117514	0.458	1.02	[1.01–1.02]	4.00×10^{-14}	Howard DM
11	*MRPL48*	rs113485090	0.040	0.32	[0.2–0.44] unit decrease	2.00×10^{-7}	Hall LS
11	*GRM5*	rs6483414	0.644	0.01	[0.0045–0.0093] unit increase	3.00×10^{-8}	Howard DM
11	*GRM5*	rs1150313	NR	6.09	z score increase	1.00×10^{-9}	Nagel M
11	*GRM5*	rs537635	0.484	1.03	[1.02–1.04]	6.00×10^{-10}	Cai N
11	*GRM5*	rs7932640	0.442	1.02	[1.019–1.027]	3.00×10^{-25}	Howard DM
11	*GRM5*	rs10501696	0.499	0.01	[0.0055–0.0103] unit decrease	7.00×10^{-11}	Howard DM
11	*GRM5 - TYR*	rs10830220	NR	6.10	z score decrease	1.00×10^{-9}	Nagel M
11	*TYR*	rs621313	0.491	0.01	[0.005–0.0098] unit increase	3.00×10^{-10}	Howard DM
11	*TYR*	rs645592	NR	5.57	z score decrease	3.00×10^{-8}	Nagel M
11	*TYR*	rs598769	0.470	0.01	[0.0034–0.0082] unit increase	9.00×10^{-7}	Howard DM
11	*AP002364.1*	rs578174	0.898	1.05	[1.03–1.07]	2.00×10^{-8}	Cai N
11	*AP002364.1*	rs578174	0.104	0.01	[0.0062–0.0136] unit decrease	3.00×10^{-7}	Howard DM
11	*CNTN5*	rs1504721	0.398	1.07	[1.04–1.1]	4.00×10^{-6}	Coleman JRI
11	*CNTN5*	rs1504722	0.397	1.14	[1.08–1.2]	9.00×10^{-6}	Coleman JRI
11	*CNTN5*	rs586533	NR	6.03	z score decrease	2.00×10^{-9}	Nagel M
11	*DYNC2H1 - AP002989.1*	rs141208948	0.021	1.07	[1.04–1.1]	3.00×10^{-6}	Coleman JRI
11	*CASP4*	rs666723	NR	0.12	[0.061–0.179] unit increase	7.00×10^{-6}	Hall LS
11	*LINC02550 - C11orf53*	rs10736455	NR	0.16	[0.082–0.238] unit decrease	4.00×10^{-6}	Hall LS
11	*DRD2 - TMPRSS5*	rs4936275	0.622	0.01	[0.0041–0.0089] unit increase	1.00×10^{-7}	Howard DM
11	*DRD2 - TMPRSS5*	rs61902811	0.632	1.03	[1.02–1.03]	4.00×10^{-39}	Howard DM
11	*ATP5MG, AP001267.5*	rs60545357	0.174	0.01	[0.0046–0.0108] unit increase	7.00×10^{-7}	Howard DM
11	*AP001993.1*	rs57344483	0.074	1.02	[1.02–1.03]	5.00×10^{-9}	Howard DM
11	*RPS27P20 - AP003500.1*	rs149415148	0.012	0.03	[0.02–0.044] unit increase	4.00×10^{-7}	Howard DM
11	*OPCML*	rs11223270	0.314	1.07	[1.04–1.1]	8.00×10^{-6}	Coleman JRI
12	*PARP11-AS1 - AC084375.1*	rs4238010	0.020	1.72		6.00×10^{-6}	Muglia P
12	*AC007207.1 - CCND2-AS1*	rs61909191	0.934	1.08	[1.04–1.11]	3.00×10^{-6}	Coleman JRI
12	*AC008012.1*	rs4625554	0.300	0.19	[0.11–0.28] unit increase	9.00×10^{-6}	GENDEP Investigators
12	*LINC02366*	rs111275535	0.060	1.13	[1.08–1.19]	5.00×10^{-6}	Coleman JRI
12	*AC022276.1 - LINC01559*	rs11055387	0.090	2.19	[1.84–2.53]	8.00×10^{-6}	GENDEP Investigators
12	*GRIN2B*	rs1457614	0.181	1.47	[1.29–1.64]	8.00×10^{-6}	GENDEP Investigators
12	*AC048352.1*	rs1706631	0.408	1.35	[1.22–1.49]	6.00×10^{-6}	GENDEP Investigators
12	*AC084816.1*	rs2466882	0.582	1.08	[1.05–1.11]	1.00×10^{-6}	Coleman JRI
12	*SOX5*	rs4074723	0.590	1.03	[1.02–1.04]	3.00×10^{-8}	Wray NR
12	*SOX5*	rs78337797	0.878	1.02	[1.016–1.03]	1.00×10^{-11}	Howard DM
12	*SOX5*	rs17487383	NR	5.50	z score decrease	4.00×10^{-8}	Nagel M
12	*AC024145.1, ITPR2*	rs10505997	0.882	0.04	[0.027–0.062] unit increase	2.00×10^{-6}	Hyde CL
12	*AC023511.1*	rs11613048	NR	0.10	[0.061–0.139] unit decrease	8.00×10^{-6}	Hall LS
12	*CPM - AC127894.1*	rs9943849	0.730	1.37	[1.20–1.56]	3.00×10^{-6}	Rietschel M
12	*AC090015.1*	rs17828731	NR	5.67	z score increase	1.00×10^{-8}	Nagel M
12	*AC093025.1 - AC090679.3*	rs56314503	0.251	1.02	[1.02–1.03]	1.00×10^{-16}	Howard DM
12	*AC093025.1 - AC090679.3*	rs4480613	NR	5.93	z score increase	3.00×10^{-9}	Nagel M
12	*AC090679.2 - AC087888.1*	rs2956278	0.234	1.04	[NR]	2.00×10^{-7}	Li X
12	*AC090679.2 - AC087888.1*	rs2956287	NR	6.57	z score decrease	5.00×10^{-11}	Nagel M
12	*AC087888.1 - AC093027.1*	rs2125716	0.765	0.03	[0.021–0.048] unit decrease	3.00×10^{-8}	Hyde CL
12	*NTS - MGAT4C*	rs1448094	NR	0.10	[0.061–0.139] unit increase	1.00×10^{-6}	Hall LS
12	*PLXNC1*	rs73228161	NR	0.19	[0.11–0.27] unit increase	6.00×10^{-6}	Hall LS
12	*SUDS3*	rs521048	0.731	0.01	[0.0042–0.0092] unit increase	5.00×10^{-7}	Howard DM
12	*SPPL3*	rs3213572	0.475	1.02	[1.016–1.024]	6.00×10^{-20}	Howard DM
12	*SPPL3*	rs3897746	0.483	0.03	[0.017–0.041] unit increase	2.00×10^{-7}	Hyde CL
12	*SPPL3*	rs1151849	NR	5.60	z score decrease	2.00×10^{-8}	Nagel M

Chr	Gene	SNP	Risk Allele Frequency	Beta	95% CI	P	First Author
12	*RPL12P33 - HNF1A-AS1*	rs77741769	NR	5.78	z score increase	8.00×10^{-9}	Nagel M
12	*ORAI1*	rs782472239	NR			9.00×10^{-7}	Wong ML
12	*LINC02824*	rs1706497	NR	5.54	z score increase	3.00×10^{-8}	Nagel M
12	*LINC02375*	rs10744304	0.630	0.15	[NR] unit decrease	9.00×10^{-6}	Terracciano A
13	*B3GLCT*	rs9530139	0.196	0.01	[0.006–0.0118] unit decrease	3.00×10^{-9}	Howard DM
13	*B3GLCT - AL161616.2*	rs1409379	0.764	1.02	[1.012–1.022]	8.00×10^{-11}	Howard DM
13	*ENOX1*	rs17538444	0.070	0.39	[0.24–0.54] unit decrease	4.00×10^{-7}	GENDEP Investigators
13	*ENOX1*	rs4143229	0.080	1.05	[1.03–1.07]	3.00×10^{-8}	Wray NR
13	*OLFM4*	rs8181889	NR	5.49	z score decrease	4.00×10^{-8}	Nagel M
13	*OLFM4*	rs12552	0.555	0.04	[0.032–0.055] unit decrease	8.00×10^{-9}	Hyde CL
13	*OLFM4*	rs12552	NR	6.37	z score increase	2.00×10^{-10}	Nagel M
13	*OLFM4*	rs12552	0.440	1.04	[1.03–1.05]	6.00×10^{-19}	Wray NR
13	*OLFM4 - LINC01065*	rs1343607	0.375	1.08	[1.05–1.11]	1.00×10^{-6}	Coleman JRI
13	*OLFM4 - LINC01065*	rs1343605	0.384	1.02	[1.019–1.028]	2.00×10^{-25}	Howard DM
13	*LINC01065*	rs2806985	NR	5.63	z score increase	2.00×10^{-8}	Nagel M
13	*LINC01065*	rs1322944	NR	5.98	z score increase	2.00×10^{-9}	Nagel M
13	*AL356295.1, AL450423.2*	rs1867368	0.317	1.03	[NR]	5.00×10^{-8}	Li X
13	*PCDH9*	rs9540720	0.500	0.03	[0.019–0.042] unit increase	9.00×10^{-7}	Hyde CL
13	*PCDH9*	rs9592461	NR	7.06	z score increase	2.00×10^{-12}	Nagel M
13	*PCDH9*	rs9592461	0.487	1.03	[1.02–1.029]	1.00×10^{-28}	Howard DM
13	*PCDH9*	rs1413573	0.492	1.03	[NR]	2.00×10^{-8}	Li X
13	*PCDH9*	rs4883777	NR	5.71	z score increase	1.00×10^{-8}	Nagel M
13	*PCDH9*	rs78643921	0.896	1.08	[1.05–1.11]	1.00×10^{-6}	Coleman JRI
13	*AL512452.1*	rs6562505	NR	5.47	z score decrease	5.00×10^{-8}	Nagel M
13	*ATXN8OS - RNU6-54P*	rs9572423	0.880	1.54		9.00×10^{-6}	Shi J
13	*LINC01068 - AL136442.1*	rs9601248	0.498	1.38	[1.25–1.52]	2.00×10^{-6}	GENDEP Investigators
13	*AL137781.1 - SPRY2*	rs9545360	0.819	1.02	[1.01–1.02]	7.00×10^{-9}	Howard DM
13	*AL137781.1 - SPRY2*	rs1755255	0.712	0.01	[0.0039–0.0089] unit increase	8.00×10^{-7}	Howard DM
13	*AL590807.1*	rs11149178	0.356	0.18	[0.1–0.26] unit decrease	7.00×10^{-6}	GENDEP Investigators
13	*LINC00430 - UBBP5*	rs139073054	NR	0.35	[0.21–0.49] unit increase	2.00×10^{-6}	Hall LS
13	*LINC00410*	rs1031396	NR	0.40	[0.22–0.58] unit decrease	5.00×10^{-6}	Hall LS
13	*STK24*	rs4772087	0.373	1.02	[1.017–1.026]	2.00×10^{-20}	Howard DM
13	*NALCN*	rs9300648	NR	0.16	[0.1–0.22] unit decrease	6.00×10^{-6}	Hall LS
13	*AL354741.1 - FAM155A*	rs1927745	0.240	0.16	[NR] unit decrease	5.00×10^{-6}	Terracciano A
13	*RPL21P107 - LINC00567*	rs4438172	0.250	0.11	[0.071–0.149] unit increase	9.00×10^{-7}	Hall LS
13	*RASA3*	rs67917624	0.027	1.16	[1.1–1.22]	1.00×10^{-6}	Coleman JRI
14	*EGLN3*	rs60225398	0.761	1.08	[1.04–1.11]	2.00×10^{-6}	Coleman JRI
14	*AL121821.2 - LRFN5*	rs61990288	0.492	1.03	[1.02–1.03]	2.00×10^{-31}	Howard DM
14	*LRFN5*	rs1900599	0.278	0.01	[0.004–0.009] unit increase	6.00×10^{-7}	Howard DM
14	*LRFN5*	rs4904738	0.430	1.03	[1.02–1.04]	3.00×10^{-9}	Wray NR
14	*TBPL2*	rs72717793	0.228	1.14	[1.08–1.19]	6.00×10^{-6}	Coleman JRI
14	*DAAM1*	rs1957408	0.491	1.15	[1.09–1.21]	2.00×10^{-6}	Coleman JRI
14	*RTN1*	rs1956373	0.256	1.02	[1.01–1.02]	6.00×10^{-12}	Howard DM
14	*KCNH5*	rs8012941	0.498	1.54	[1.35–1.72]	4.00×10^{-6}	GENDEP Investigators
14	*ESR2, SYNE2*	rs915057	0.580	1.03	[1.02–1.04]	8.00×10^{-10}	Wray NR
14	*ESR2*	rs1152578	0.564	1.02	[1.01–1.02]	4.00×10^{-12}	Howard DM
14	*GPHN*	rs8020095	0.170			3.00×10^{-6}	Hek K
14	*PLEK2*	rs9323497	0.050			4.00×10^{-9}	Amare AT
14	*AREL1*	rs1045430	0.521	1.02	[1.02–1.03]	4.00×10^{-25}	Howard DM
14	*YLPM1*	rs12889665	0.538	1.03	[1.02–1.04]	3.00×10^{-8}	Cai N
14	*YLPM1*	rs10148293	0.539	0.01	[0.0038–0.0086] unit increase	1.00×10^{-7}	Howard DM
14	*ADCK1 - FXNP1*	rs4903685	0.275	1.07	[1.04–1.1]	8.00×10^{-6}	Coleman JRI
14	*AL356807.1*	rs144213213	0.981	1.09	[1.05–1.12]	2.00×10^{-7}	Coleman JRI
14	*AL163642.2 - AL357172.1*	rs79306025	NR	0.44	[0.26–0.62] unit increase	2.00×10^{-6}	Hall LS
14	*PRIMA1, PRIMA1*	rs8008104	0.095	1.07	[1.04–1.1]	6.00×10^{-6}	Coleman JRI
14	*LINC02304*	rs60716536	NR	0.13	[0.071–0.189] unit decrease	3.00×10^{-6}	Hall LS
14	*CDC42BPB*	rs117911063	0.031	0.02	[0.012–0.026] unit increase	4.00×10^{-7}	Howard DM
14	*RNU7-160P - AL139300.2*	rs10149470	0.513	1.02	[1.02–1.02]	7.00×10^{-22}	Howard DM
14	*RNU7-160P - AL139300.2*	rs10149470	0.510	1.03	[1.02–1.04]	3.00×10^{-9}	Wray NR
14	*KLC1, AL139300.1*	rs4906349	0.311	0.01	[0.0039–0.0089] unit decrease	5.00×10^{-7}	Howard DM
14	*TDRD9*	rs61997596	0.191	1.04	[1.02–1.05]	4.00×10^{-8}	Cai N
15	*AC087490.1 - PWRN4*	rs1380551	0.150	0.18	[0.1–0.26] unit decrease	4.00×10^{-7}	Hall LS
15	*FMN1 - TMCO5B*	rs974379	0.075	1.29	[1.17–1.4]	8.00×10^{-6}	GENDEP Investigators
15	*AC021351.1, LINC02853*	rs10520045	0.270	1.32	[1.17–1.50]	7.00×10^{-6}	Rietschel M
15	*AC087516.2 - COX6CP4*	rs17705482	NR	0.11	[0.051–0.169] unit decrease	9.00×10^{-6}	Hall LS
15	*AC087283.1 - AC068875.1*	rs8037355	0.444	1.02	[1.02–1.02]	2.00×10^{-18}	Howard DM
15	*AC087283.1 - AC068875.1*	rs8025231	0.428	0.04	[0.024–0.047] unit increase	1.00×10^{-8}	Hyde CL
15	*AC087283.1 - AC068875.1*	rs8025231	0.430	1.03	[1.02–1.04]	2.00×10^{-12}	Wray NR

Chr	Gene	SNP	Risk Allele Frequency	Beta	95% CI	P	First Author
15	*AC068875.1*	rs1457257	0.366	1.04	[NR]	1.00×10^{-8}	Li X
15	*SEMA6D, AC023905.1*	rs34488670	0.211	1.02	[1.01–1.02]	5.00×10^{-13}	Howard DM
15	*SEMA6D*	rs117319730	NR	5.54	z score decrease	3.00×10^{-8}	Nagel M
15	*SEMA6D*	rs72733826	NR	5.91	z score increase	3.00×10^{-9}	Nagel M
15	*SHC4*	rs8023445				2.00×10^{-6}	Aragam N
15	*SHC4, AC091073.2*	rs11070668	NR	1.41	[1.22–1.61]	4.00×10^{-6}	Howard DM
15	*MIR4713HG, TNFAIP8L3*	rs150406602	0.029	1.14	[1.09–1.2]	1.00×10^{-6}	Coleman JRI
15	*LYSMD2*	rs2414116	0.462	1.07	[1.04–1.1]	3.00×10^{-6}	Coleman JRI
15	*RORA*	rs12912233	0.460	0.15	[NR] unit increase	6.00×10^{-7}	Terracciano A
15	*ITGA11*	rs7174755	0.369	0.20	[0.13–0.28] unit decrease	3.00×10^{-7}	GENDEP Investigators
15	*ITGA11*	rs7174755	0.630	1.17	[1.11–1.23]	9.00×10^{-7}	GENDEP Investigators
15	*LINGO1*	rs7170398	NR	5.62	z score increase	2.00×10^{-8}	Nagel M
15	*AC105133.1, LINGO1*	rs11629621	NR	5.48	z score decrease	4.00×10^{-8}	Nagel M
15	*NTRK3-AS1 - MRPL46*	rs28541419	0.231	0.01	[0.0051–0.0105] unit decrease	3.00×10^{-8}	Howard DM
15	*AC024651.3 - ARRDC4*	rs74031748	NR	0.19	[0.11–0.27] unit decrease	7.00×10^{-6}	Hall LS
15	*LYSMD4 - DNM1P46*	rs6598266	0.143	0.25	[0.15–0.36] unit increase	3.00×10^{-6}	GENDEP Investigators
16	*TSC2*	rs8050755	NR	0.11	[0.071–0.149] unit increase	9.00×10^{-6}	Hall LS
16	*AC009065.6, PKD1*	rs74002781	NR	0.21	[0.11–0.31] unit decrease	7.00×10^{-6}	Hall LS
16	*RBFOX1, AC009135.1*	rs1811320	0.582	1.08	[1.05–1.11]	1.00×10^{-6}	Coleman JRI
16	*RBFOX1*	rs8063603	0.350	1.03	[1.02–1.04]	7.00×10^{-9}	Wray NR
16	*RBFOX1*	rs7193263	NR	6.48	z score decrease	9.00×10^{-11}	Nagel M
16	*RBFOX1*	rs7193263	0.332	1.02	[1.02–1.03]	4.00×10^{-19}	Howard DM
16	*RBFOX1*	rs7198928	0.616	1.02	[1.018–1.027]	4.00×10^{-23}	Howard DM
16	*RBFOX1*	rs7198928	0.620	1.03	[1.02–1.04]	1.00×10^{-8}	Wray NR
16	*RBFOX1*	rs3785236	0.755	0.04	[0.023–0.05] unit increase	9.00×10^{-7}	Hyde CL
16	*RBFOX1*	rs3785234	0.624	1.04	[NR]	2.00×10^{-8}	Li X
16	*RBFOX1*	rs2191130	NR	6.10	z score increase	1.00×10^{-9}	Nagel M
16	*SNX29*	rs830716	0.714	0.01	[0.0041–0.0091] unit increase	5.00×10^{-7}	Howard DM
16	*SHISA9*	rs7200826	0.255	1.03	[1.022–1.032]	3.00×10^{-28}	Howard DM
16	*SHISA9*	rs7200826	0.250	1.03	[1.02–1.04]	2.00×10^{-8}	Wray NR
16	*U91319.1 - U95743.1*	rs56887639	0.274	1.02	[1.01–1.02]	6.00×10^{-13}	Howard DM
16	*U91319.1 - U95743.1*	rs12932870	0.187	0.01	[0.0053–0.0111] unit increase	7.00×10^{-8}	Howard DM
16	*METTL9, METTL9*	rs11646401	0.442	1.03	[1.02–1.04]	4.00×10^{-8}	Cai N
16	*METTL9, METTL9*	rs1478461	0.662	0.01	[0.0042–0.009] unit increase	1.00×10^{-7}	Howard DM
16	*METTL9, METTL9*	rs12923444	0.438	1.02	[1.02–1.03]	2.00×10^{-24}	Howard DM
16	*AC127459.2 - AC127459.1*	rs6497642	0.679	1.15	[1.09–1.21]	4.00×10^{-6}	Coleman JRI
16	*AC007861.1*	rs35888008	NR	0.09	[0.051–0.129] unit increase	4.00×10^{-6}	Hall LS
16	*AC007861.1*	rs35888008	NR	0.12	[0.061–0.179] unit increase	6.00×10^{-6}	Hall LS
16	*LINC01571 - C16orf97*	rs1486437	NR	0.13	[0.071–0.189] unit decrease	9.00×10^{-6}	Hall LS
16	*CASC22, AC007333.1*	rs10521261	0.801	0.03	[0.018–0.05] unit increase	3.00×10^{-7}	Hyde CL
16	*LINC02141 - AC009081.2*	rs117225626	0.015	1.15	[1.09–1.2]	5.00×10^{-7}	Coleman JRI
16	*PMFBP1 - AC009075.1*	rs11643192	0.410	1.03	[1.02–1.04]	3.00×10^{-8}	Wray NR
16	*ZFHX3, LINC01568*	rs12446956	0.130	1.22	[NR]	1.00×10^{-6}	Wray NR
16	*AC106733.1 - MON1B*	rs2342343	0.306	1.07	[1.04–1.1]	6.00×10^{-6}	Coleman JRI
16	*CDH13*	rs10514585	0.310	0.15	[NR] unit increase	5.00×10^{-6}	Terracciano A
16	*AC092723.2 - AC135012.1*	rs8050326	0.440	1.45	[1.26–1.76]	3.00×10^{-7}	Power RA
16	*ZCCHC14*	rs9646303	NR			5.00×10^{-6}	Sullivan PF
17	*AC005291.2, MYH13*	rs3744550	NR			4.00×10^{-6}	Wong ML
17	*AC015908.7 - RN7SL601P*	rs117183159	0.949	1.07	[1.04–1.1]	9.00×10^{-6}	Coleman JRI
17	*PIPOX*	rs75581564	0.117	1.03	[1.018–1.032]	3.00×10^{-13}	Howard DM
17	*PIPOX*	rs8066520	0.850	0.04	[0.022–0.054] unit decrease	4.00×10^{-6}	Hyde CL
17	*CRYBA1*	rs17727765	0.080	1.05	[1.04–1.07]	9.00×10^{-9}	Wray NR
17	*ASIC2*	rs34187563	0.405	0.01	[0.004–0.0088] unit decrease	3.00×10^{-7}	Howard DM
17	*AC015911.11*	rs8070473	0.320	0.16	[NR] unit decrease	2.00×10^{-6}	Terracciano A
17	*KRTAP4-17P - KRTAP9-1*	rs56294834	0.021	1.13	[1.07–1.18]	8.00×10^{-6}	Coleman JRI
17	*AC100793.3, CNTNAP1*	rs142029931	NR			1.00×10^{-6}	Wong ML
17	*SUPT4H1, TSPOAP1-AS1, AC004687.2*	rs7222143	0.445	1.07	[1.04–1.1]	7.00×10^{-6}	Coleman JRI
17	*WIPI1, AC007780.1*	rs12943181	0.280	0.01	[0.0046–0.0096] unit decrease	6.00×10^{-8}	Howard DM
17	*UNC13D*	rs150952348	NR			7.00×10^{-7}	Wong ML
17	*RNF213*	rs35864932	0.475	1.07	[1.04–1.1]	4.00×10^{-6}	Coleman JRI
17	*BAIAP2*	rs55748329	NR	6.10	z score increase	1.00×10^{-9}	Nagel M
17	*FASN*	rs200897153	NR			2.00×10^{-6}	Wong ML
18	*AP005230.1*	rs56039004	0.070	1.15	[1.09–1.2]	8.00×10^{-7}	Coleman JRI
18	*AP005061.1 - RNU6-340P*	rs9964540	0.009	1.08	[1.04–1.11]	3.00×10^{-6}	Coleman JRI
18	*PIEZO2*	rs264272	0.520	1.14	[1.09–1.2]	4.00×10^{-6}	GENDEP Investigators
18	*PIEZO2*	rs264272	0.487	0.17	[0.099–0.243] unit decrease	3.00×10^{-6}	GENDEP Investigators
18	*AC115100.1 - AC090506.1*	rs972697	0.206	1.07	[1.04–1.1]	8.00×10^{-6}	Coleman JRI
18	*ASXL3 - NOL4*	rs10460050	0.421	1.07	[1.04–1.1]	9.00×10^{-6}	Coleman JRI

Chr	Gene	SNP	Risk Allele Frequency	Beta	95% CI	P	First Author
18	*CELF4*	rs4799936	NR	5.64	z score decrease	2.00×10^{-8}	Nagel M
18	*CELF4*	rs11877758	0.311	0.01	[0.0042–0.0092] unit increase	1.00×10^{-7}	Howard DM
18	*CELF4*	rs12967855	0.329	1.03	[1.02–1.05]	2.00×10^{-9}	Cai N
18	*CELF4*	rs12967855	0.330	1.02	[1.016–1.026]	1.00×10^{-19}	Howard DM
18	*AC009899.1*	rs11665070	NR	6.04	z score decrease	2.00×10^{-9}	Nagel M
18	*AC009899.1*	rs1427027	NR	5.63	z score increase	2.00×10^{-8}	Nagel M
18	*RNU6-706P - MIR924HG*	rs57679688	0.263	0.01	[0.0044–0.0094] unit increase	3.00×10^{-7}	Howard DM
18	*MIR924HG*	rs62099069	0.580	1.03	[1.02–1.04]	1.00×10^{-8}	Wray NR
18	*MIR924HG*	rs2163544	0.420	0.03	[0.019–0.043] unit decrease	2.00×10^{-6}	Hyde CL
18	*SYT4 - AC022743.1*	rs12457996	0.770	1.15	[NR]	6.00×10^{-6}	Wray NR
18	*DCC*	rs149735550	NR	5.53	z score increase	3.00×10^{-8}	Nagel M
18	*DCC*	rs62097899	NR	6.15	z score increase	8.00×10^{-10}	Nagel M
18	*DCC*	rs11663393	0.450	1.03	[1.02–1.04]	2.00×10^{-8}	Wray NR
18	*DCC*	rs4277413	NR	6.77	z score decrease	1.00×10^{-11}	Nagel M
18	*DCC*	rs7505145	NR	5.62	z score decrease	2.00×10^{-8}	Nagel M
18	*DCC*	rs7227069	0.433	1.02	[1.02–1.029]	2.00×10^{-28}	Howard DM
18	*DCC*	rs12968428	NR	7.07	z score increase	2.00×10^{-12}	Nagel M
18	*DCC*	rs8099160	NR	7.64	z score increase	2.00×10^{-14}	Nagel M
18	*DCC*	rs17410557	0.391	0.01	[0.0035–0.0083] unit increase	8.00×10^{-7}	Howard DM
18	*DCC*	rs1431181	NR	6.96	z score increase	3.00×10^{-12}	Nagel M
18	*DCC*	rs8089865	NR	6.62	z score increase	3.00×10^{-11}	Nagel M
18	*RAB27B*	rs62091461	0.773	1.02	[1.02–1.03]	2.00×10^{-14}	Howard DM
18	*RAB27B*	rs1833288	0.720	1.03	[1.02–1.04]	3.00×10^{-8}	Wray NR
18	*CCDC68 - LINC01929*	rs12966052	0.820	1.02	[1.01–1.02]	4.00×10^{-11}	Howard DM
18	*CCDC68 - LINC01929*	rs12963231	NR	5.53	z score increase	3.00×10^{-8}	Nagel M
18	*RNA5SP459 - TCF4*	rs4801146	0.492	0.01	[0.0039–0.0087] unit increase	1.00×10^{-7}	Howard DM
18	*TCF4*	rs1261070	NR	5.70	z score decrease	1.00×10^{-8}	Nagel M
18	*TCF4*	rs12967143	0.702	0.01	[0.0045–0.0095] unit decrease	6.00×10^{-8}	Howard DM
18	*TCF4*	rs12967143	0.302	1.03	[1.02–1.03]	2.00×10^{-27}	Howard DM
18	*TCF4*	rs12958048	0.330	1.03	[1.02–1.04]	4.00×10^{-11}	Wray NR
18	*TCF4*	rs1452788	NR	5.45	z score decrease	5.00×10^{-8}	Nagel M
18	*TCF4*	rs4801157	NR	5.50	z score increase	4.00×10^{-8}	Nagel M
18	*TCF4*	rs1452787	0.274	0.01	[0.0043–0.0093] unit increase	2.00×10^{-7}	Howard DM
18	*AC022031.2*	rs8097498	0.387	1.03	[1.02–1.04]	1.00×10^{-8}	Cai N
18	*AC006305.2*	rs1792737	NR	0.10	[0.061–0.139] unit decrease	2.00×10^{-6}	Hall LS
18	*CCBE1*	rs11152166	0.580	1.41	[1.22–1.61]	3.00×10^{-6}	Power RA
18	*LINC01544 - RNF152*	rs4941058	NR	0.13	[0.071–0.189] unit decrease	1.00×10^{-6}	Hall LS
18	*SERPINB13*	rs6567380	0.023	1.14	[1.08–1.19]	2.00×10^{-6}	Coleman JRI
18	*AC114689.3*	rs17077540	0.110	1.61		2.00×10^{-7}	Shi J
18	*AC021594.2 - KCNG2*	rs7241572	0.201	1.02	[1.015–1.026]	3.00×10^{-13}	Howard DM
19	*CATSPERD*	rs79696139	0.051	1.07	[1.04–1.1]	9.00×10^{-6}	Coleman JRI
19	*MARCHF2*	rs117621296	NR	0.31	[0.17–0.45] unit decrease	9.00×10^{-6}	Hall LS
19	*ADGRE2*	rs112610420	NR			1.00×10^{-6}	Wong ML
19	*ANO8*	rs143696449	NR			5.00×10^{-7}	Wong ML
19	*ZNF536*	rs33431	0.614	1.01	[1.008–1.016]	5.00×10^{-8}	Howard DM
19	*ZNF536*	rs33435	0.413	0.03	[0.015–0.039] unit decrease	2.00×10^{-6}	Hyde CL
19	*ANKRD27*	rs17692896	0.074	0.44	[0.27–0.61] unit decrease	7.00×10^{-7}	GENDEP Investigators
19	*KCTD15 - AC008556.1*	rs2546057	0.520	1.18	[1.12–1.24]	3.00×10^{-7}	GENDEP Investigators
19	*KCTD15 - AC008556.1*	rs398426	0.259	0.25	[0.14–0.36] unit decrease	5.00×10^{-6}	GENDEP Investigators
19	*LILRA1, LILRA1, LILRA1, LILRA1*	rs201483250	NR			1.00×10^{-6}	Wong ML
19	*TMEM150B*	rs201935337	NR			5.00×10^{-11}	Wong ML
19	*NLRP9 - RFPL4A*	rs17634917	0.940	1.37	[1.23–1.51]	3.00×10^{-6}	GENDEP Investigators
19	*NLRP9 - RFPL4A*	rs17634917	0.057	0.38	[0.21–0.54] unit decrease	6.00×10^{-6}	GENDEP Investigators
20	*PLCG1*	rs2228246	0.163	0.04	[0.026–0.058] unit increase	3.00×10^{-7}	Hyde CL
20	*ZHX3 - ADI1P1*	rs6065333	0.150	1.04	[NR]	9.00×10^{-8}	Li X
20	*EMILIN3 - AL031667.1*	rs143186028	0.178	1.03	[1.019–1.03]	7.00×10^{-18}	Howard DM
20	*SLC12A5*	rs12624433	0.258	1.02	[1.014–1.024]	2.00×10^{-14}	Howard DM
20	*NCOA5 - RPL13P2*	rs6131010	0.740	0.01	[0.0048–0.0098] unit decrease	7.00×10^{-8}	Howard DM
20	*STAU1*	rs6066975	NR	5.47	z score decrease	4.00×10^{-8}	Nagel M
20	*AL035685.1, KCNB1*	rs3787317	NR	6.12	z score increase	9.00×10^{-10}	Nagel M
20	*KCNB1, AL035685.1*	rs6019826	NR	5.74	z score increase	9.00×10^{-9}	Nagel M
20	*KCNG1*	rs6096232	0.061	1.14	[1.08–1.2]	5.00×10^{-6}	Coleman JRI
20	*LINC01718 - CDH4*	rs143502921	0.181	0.01	[0.0057–0.0123] unit increase	1.00×10^{-7}	Howard DM
21	*AP000961.1 - AP000474.2*	rs2828520	0.310	1.35		4.00×10^{-7}	Shi J
22	*CLDN5 - LINC00895*	rs8140097	NR	0.12	[0.081–0.159] unit increase	2.00×10^{-6}	Hall LS
22	*RSPH14*	rs3788338	0.746	1.08	[1.05–1.11]	3.00×10^{-7}	Coleman JRI
22	*KIAA1671*	rs5760842	NR	0.09	[0.051–0.129] unit decrease	2.00×10^{-6}	Hall LS
22	*LARGE1*	rs5754682	0.874	0.04	[0.024–0.06] unit decrease	1.00×10^{-6}	Hyde CL

Chr	Gene	SNP	Risk Allele Frequency	Beta	95% CI	P	First Author
22	*LINC01643 - AL021877.1*	rs1034394	0.339	0.21	[0.12–0.3] unit increase	2.00×10^{-6}	GENDEP Investigators
22	*RBX1 - AL080243.3*	rs9611460	NR	5.60	z score decrease	2.00×10^{-8}	Nagel M
22	*AL080243.2 - MIR1281*	rs9623320	0.304	1.04	[NR]	4.00×10^{-8}	Li X
22	*AL080243.2 - MIR1281*	rs5995992	0.285	1.03	[1.03–1.04]	3.00×10^{-35}	Howard DM
22	*L3MBTL2, L3MBTL2-AS1*	rs5758265	0.280	1.03	[1.02–1.04]	8.00×10^{-9}	Wray NR
22	*L3MBTL2, L3MBTL2-AS1*	rs2179744	0.718	0.04	[0.022–0.049] unit decrease	6.00×10^{-11}	Hyde CL
22	*MEI1*	rs17377987	0.063	1.15	[1.09–1.21]	3.00×10^{-7}	Coleman JRI
22	*WBP2NL, SLC25A5P1*	rs34071114	0.046	1.08	[1.05–1.11]	4.00×10^{-7}	Coleman JRI
22	*Z85994.2 - LINC01656*	rs5766499	0.662	1.14	[1.08–1.2]	8.00×10^{-6}	Coleman JRI
22	*PRR5-ARHGAP8, ARHGAP8*	rs140395831	NR			3.00×10^{-10}	Wong ML
22	*PHF21B - NUP50-DT*	rs140555	NR	0.09	[0.051–0.129] unit decrease	2.00×10^{-6}	Hall LS
22	*TUBGCP6*	rs5771109	0.599	0.03	[0.015–0.039] unit decrease	7.00×10^{-6}	Hyde CL
