# Supplementary material for: Prediction of Probable Major Depressive Disorder in the Taiwan Biobank: An Integrated Machine Learning and Genome-Wide Analysis Approach
Source: J Pers Med. 2021 Jun 24;11(7):597. doi: 10.3390/jpm11070597 (PMC8308113; doi:10.3390/jpm11070597)
Supplement: Supplementary file 1 [file jpm-11-00597-s001.zip › jpm-1229212-Supplementary/Supp-Table-S8.pdf]

Table S8. Nearby SNPs between the Taiwan Biobank and previous reports from the NHGRI-EBI GWAS Catalog.

Taiwan Biobank					GWAS Catalog					
**CHR**	**POS**	**Gene**	**SNP**	**Region**	**CHR**	**POS**	**Gene**	**SNP**	**Region**	**First Author**
5	11,975,620	*CTNND2-RNU6-679P*	rs12516830	intergenic	5	10,806,645	*DAP-CTNND2*	rs6893200	intergenic	Hall LS
					5	10,820,731	*DAP-CTNND2*	rs2964802	intergenic	Hall LS
					5	10,820,731	*DAP-CTNND2*	rs2964802	intergenic	Hall LS
13	110,591,389	*RAB20-NAXD*	rs9559849	intergenic	13	110,796,311	*RPL21P107-LINC00567*	rs4438172	intergenic	Hall LS

CHR = chromosome, POS = position.
