# Supplementary material for: Prediction of Probable Major Depressive Disorder in the Taiwan Biobank: An Integrated Machine Learning and Genome-Wide Analysis Approach
Source: J Pers Med. 2021 Jun 24;11(7):597. doi: 10.3390/jpm11070597 (PMC8308113; doi:10.3390/jpm11070597)
Supplement: Supplementary file 1 [file jpm-11-00597-s001.zip › jpm-1229212-Supplementary/Suppl Figure S1.pdf]

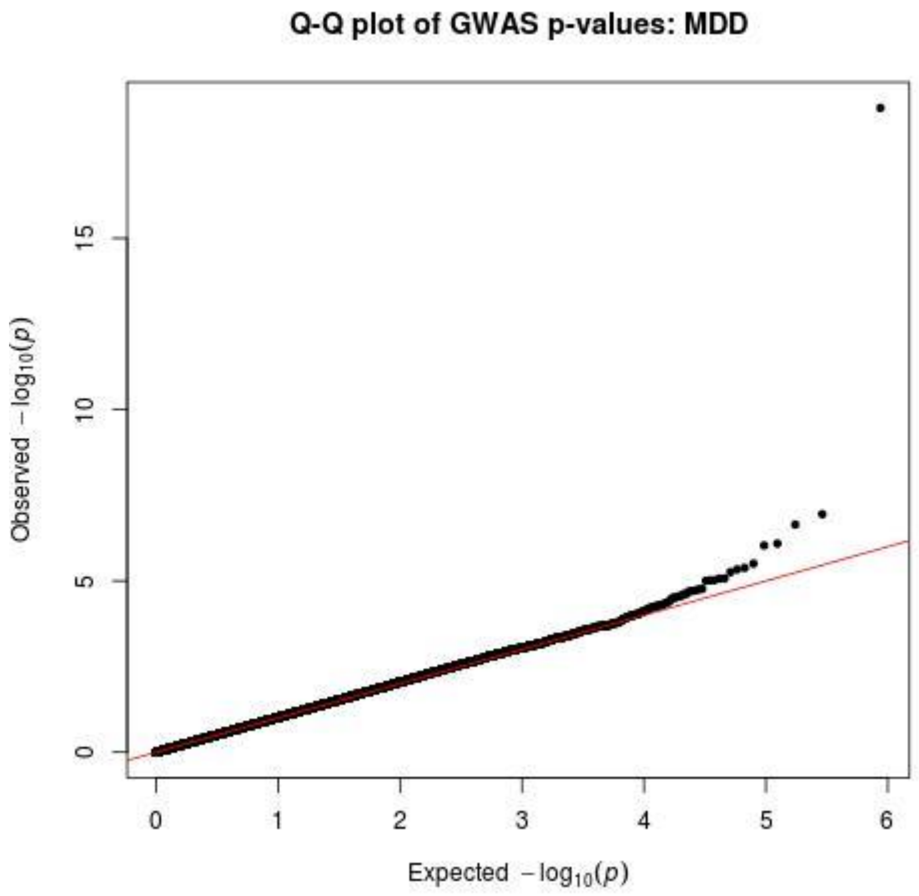

Supplementary Fig. S1. The quantile-quantile (Q-Q) plot of probable major depressive disorder (MDD) results. The plot illustrates expected and observed distributions of P-values from the genome-wide association study of probable MDD.
