# Supplementary material for: Prediction of Probable Major Depressive Disorder in the Taiwan Biobank: An Integrated Machine Learning and Genome-Wide Analysis Approach
Source: J Pers Med. 2021 Jun 24;11(7):597. doi: 10.3390/jpm11070597 (PMC8308113; doi:10.3390/jpm11070597)
Supplement: Supplementary file 1 [file jpm-11-00597-s001.zip › jpm-1229212-Supplementary/Suppl Figure S2.pdf]

(A) Logistic ridge regression vs. SVM.

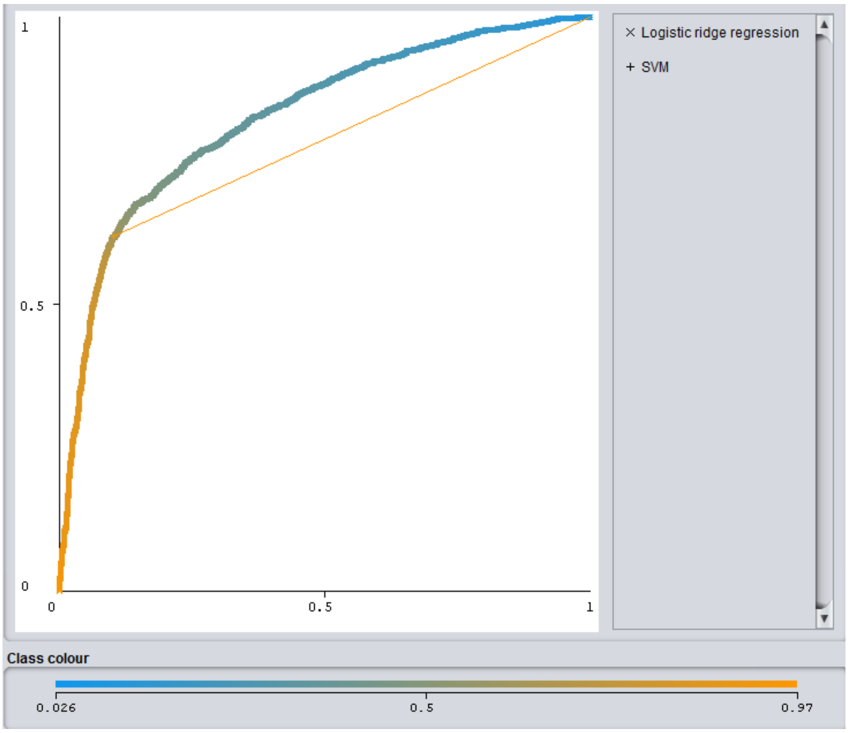

(B) Logistic ridge regression vs. C4.5 decision tree.

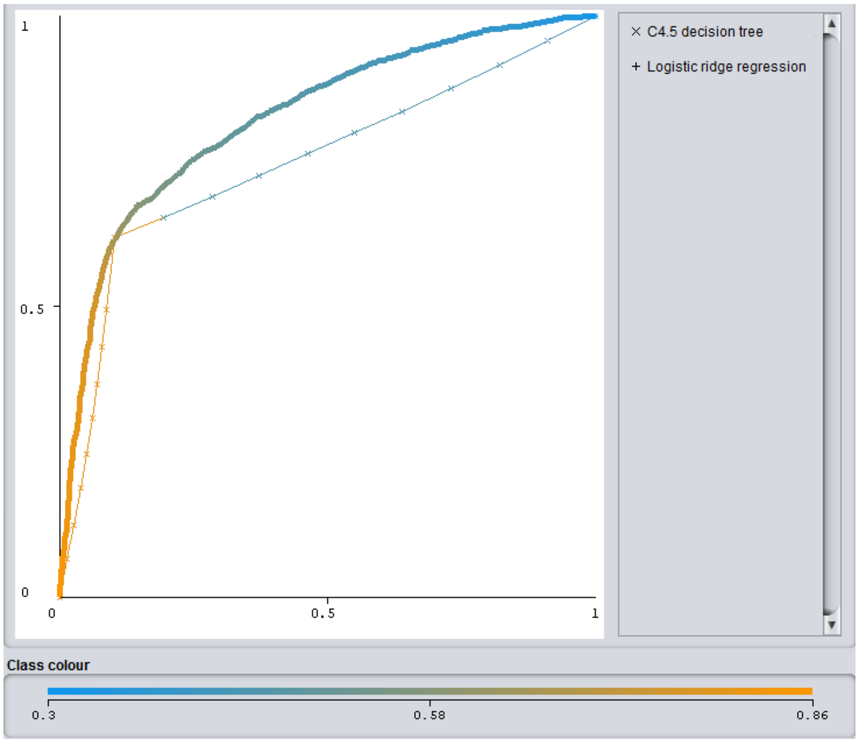

(C) Logistic ridge regression vs. LogitBoost.

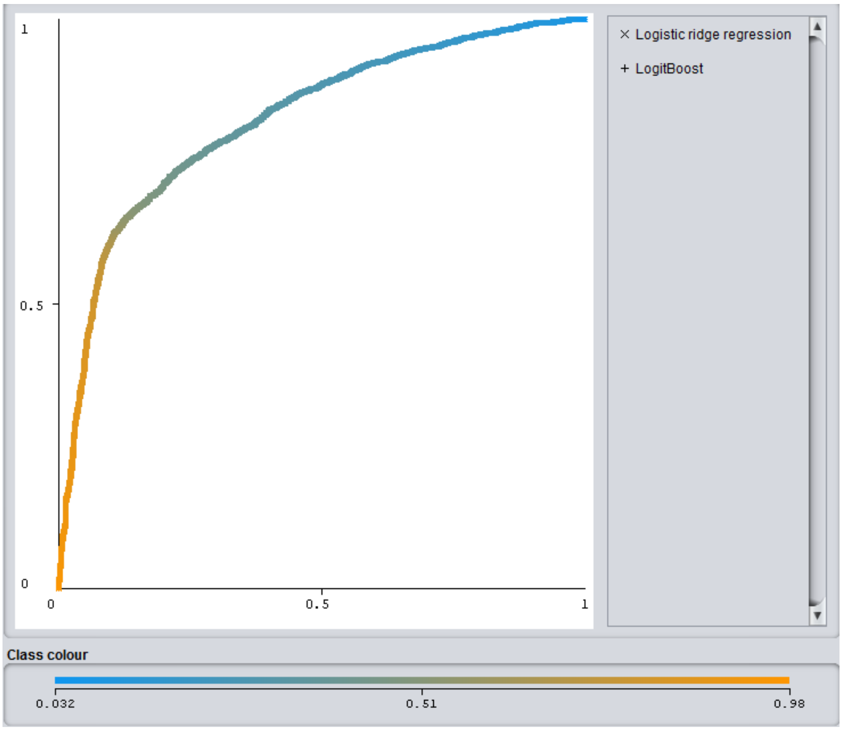

(D) Logistic ridge regression vs. Random forests.

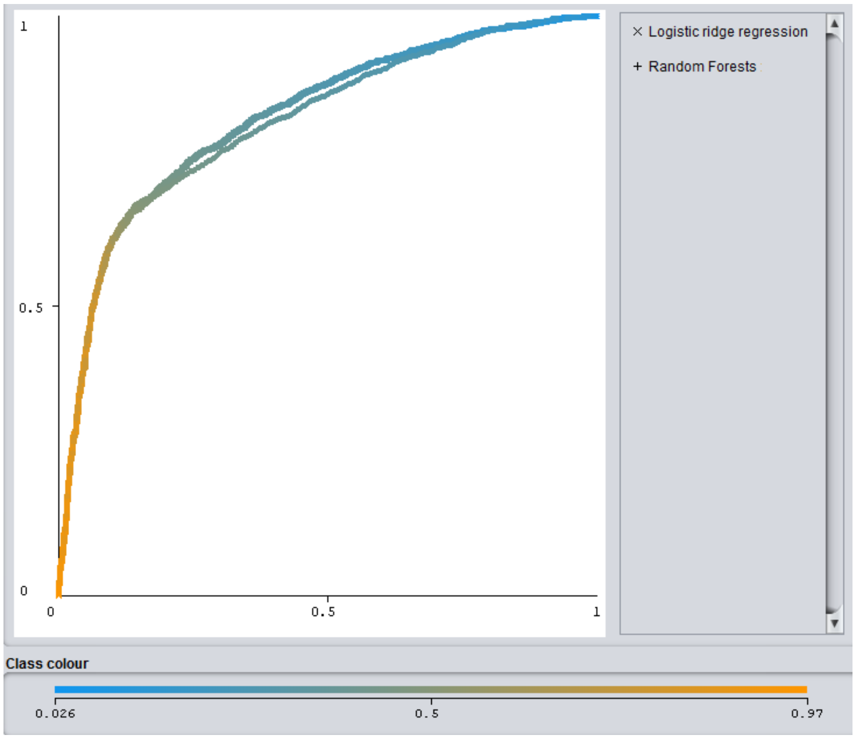

Figure S2. Plots of receiver operating characteristic (ROC) curves using random undersampling. (A) ROC curves representing logistic ridge regression (the upper curve) and support vector machine (SVM) (the lower curve); (B) ROC curves representing logistic ridge regression (the upper curve) and C4.5 decision tree (the lower curve); (C) ROC curves representing logistic ridge regression and LogitBoost (Note that two curves were overlaid.); (D) ROC curves representing logistic ridge regression (the upper curve) and random forests (the lower curve). The vertical axis represents the true positive rate, and the horizontal axis represents the false positive rate. The "class colour" indicator near the bottom represents the threshold value for each instance. While the darker brown color indicates the higher threshold value, the darker blue color indicates the lower threshold value.
