# Supplementary material for: Prediction of Probable Major Depressive Disorder in the Taiwan Biobank: An Integrated Machine Learning and Genome-Wide Analysis Approach
Source: J Pers Med. 2021 Jun 24;11(7):597. doi: 10.3390/jpm11070597 (PMC8308113; doi:10.3390/jpm11070597)
Supplement: Supplementary file 1 [file jpm-11-00597-s001.zip › jpm-1229212-Supplementary/Suppl Figure S3.pdf]

(A) Random forests vs. Logistic ridge regression.

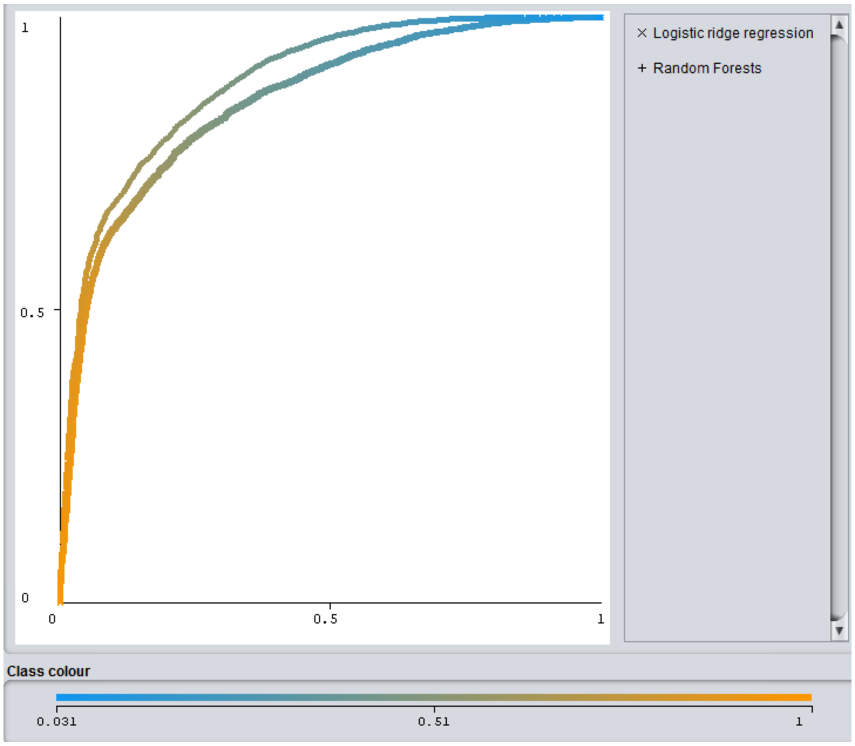

(B) Random forests vs. SVM.

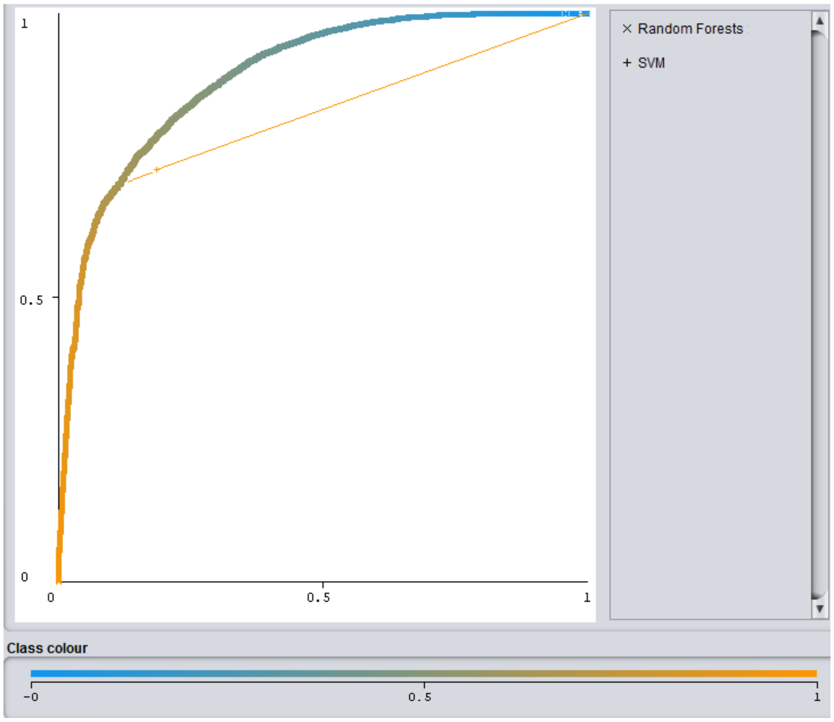

(C) Random forests vs. C4.5 decision tree.

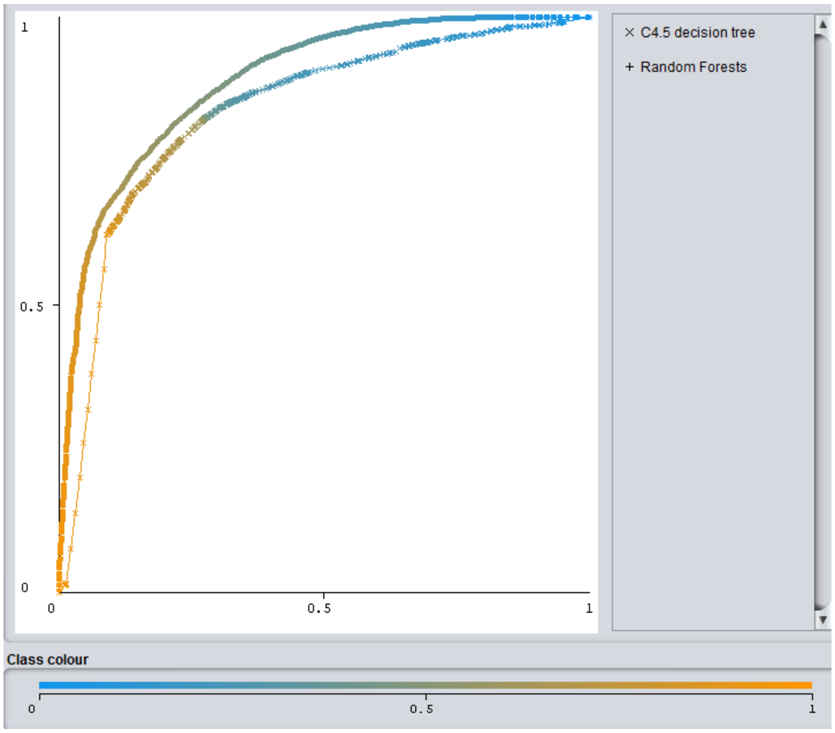

(D) Random forests vs. LogitBoost.

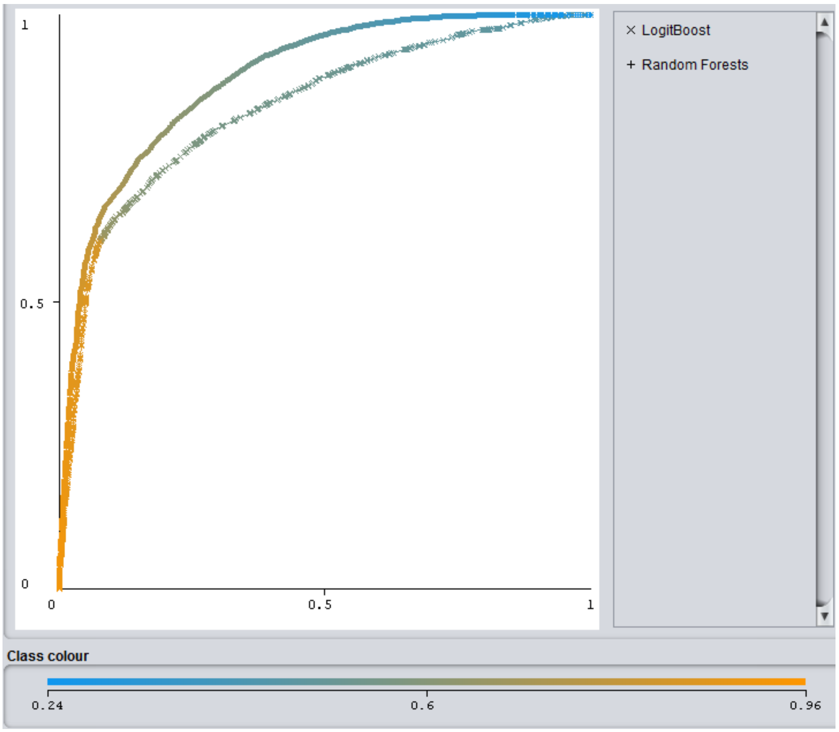

Figure S3. Plots of receiver operating characteristic (ROC) curves using synthetic minority oversampling. (A) ROC curves representing random forests (the upper curve) and logistic ridge regression (the lower curve); (B) ROC curves representing random forests (the upper curve) and support vector machine (SVM) (the lower curve); (C) ROC curves representing random forests (the upper curve) and C4.5 decision tree (the lower curve); (D) ROC curves representing random forests (the upper curve) and LogitBoost (the lower curve). The vertical axis represents the true positive rate, and the horizontal axis represents the false positive rate. The "class colour" indicator near the bottom represents the threshold value for each instance. While the darker brown color indicates the higher threshold value, the darker blue color indicates the lower threshold value.
